# Therapeutic regulation of the NLRP3 inflammasome in chronic inflammatory diseases

**DOI:** 10.1007/s12272-021-01307-9

**Published:** 2021-02-03

**Authors:** Jin Kyung Seok, Han Chang Kang, Yong-Yeon Cho, Hye Suk Lee, Joo Young Lee

**Affiliations:** BK21 PLUS Team, College of Pharmacy, The Catholic University of Korea, Bucheon, 14662 Republic of Korea

**Keywords:** Innate immunity, Inflammation, Pattern recognition receptors, Drug development, Small molecule inhibitors

## Abstract

Inflammasomes are cytosolic pattern recognition receptors that recognize pathogen-associated molecular patterns (PAMPs) and danger-associated molecular patterns (DAMPs) derived from invading pathogens and damaged tissues, respectively. Upon activation, the inflammasome forms a complex containing a receptor protein, an adaptor, and an effector to induce the autocleavage and activation of procaspase-1 ultimately culminating in the maturation and secretion of IL-1β and IL-18 and pyroptosis. Inflammasome activation plays an important role in host immune responses to pathogen infections and tissue repair in response to cellular damage. The NLRP3 inflammasome is a well-characterized pattern recognition receptor and is well known for its critical role in the regulation of immunity and the development and progression of various inflammatory diseases. In this review, we summarize recent efforts to develop therapeutic applications targeting the NLRP3 inflammasome to cure and prevent chronic inflammatory diseases. This review extensively discusses NLRP3 inflammasome-related diseases and current development of small molecule inhibitors providing beneficial information on the design of therapeutic strategies for NLRP3 inflammasome-related diseases. Additionally, small molecule inhibitors are classified depending on direct or indirect targeting mechanism to describe the current status of the development of pharmacological inhibitors.

## Introduction

Inflammasomes are multiprotein complexes formed to mediate host immune responses to pathogen infections and tissue repair in response to cellular damage (Takeuchi and Akira 2010; Kelley et al. 2019). Inflammasomes generally consist of a receptor protein and an effector with or without an adaptor. Receptor proteins include nucleotide-binding domain-like receptors (NLRs), such as NLRP1, NLRP3, NLRC4, absent in melanoma 2-like receptors (ALRs), such as AIM2, and pyrin. As pattern recognition receptors (PRRs), inflammasomes respond to a wide range of stimuli, including pathogen-associated molecular patterns (PAMPs) and danger-associated molecular patterns (DAMPs), to form a complex containing a receptor protein with the adaptor apoptosis-associated speck-like protein containing a caspase-recruitment domain (ASC) and procaspase-1. Stimulation leads to the cleavage and autoactivation of procaspase-1, which induces the maturation and secretion of proinflammatory cytokines, such as IL-1β and IL-18, and pyroptosis, an inflammatory form of programmed cell death (Fink and Cookson 2006). Extensive studies investigated the role of the inflammasome in immunity and as a significant pathological factor in chronic diseases (Menu and Vince 2011; Rubartelli 2012). NLRP3 inflammasome is one of the most well-documented inflammasome PRRs in terms of its role in the regulation of immunity and inflammatory diseases.

## Activation of NLRP3 inflammasome by exogenous and endogenous danger signals

NLRP3 inflammasome activation generally occurs in two steps: priming and activation. The priming step functions to upregulate the expression of inflammasome components, such as NLRP3, procaspase-1, and pro-IL-1β. This transcriptional upregulation is caused by other PRRs, such as Toll-like receptors (TLRs) and nucleotide-binding oligomerization domain-containing protein-2 (NOD2), at sites of infection (Franchi et al. 2009; Bauernfeind et al. 2009). Priming can also be caused by inflammatory cytokines, such as tumor necrosis factor (TNF)-α and IL-1β, which activate NF-κB and gene transcription (Xing et al. 2017).

**Fig. 1:**
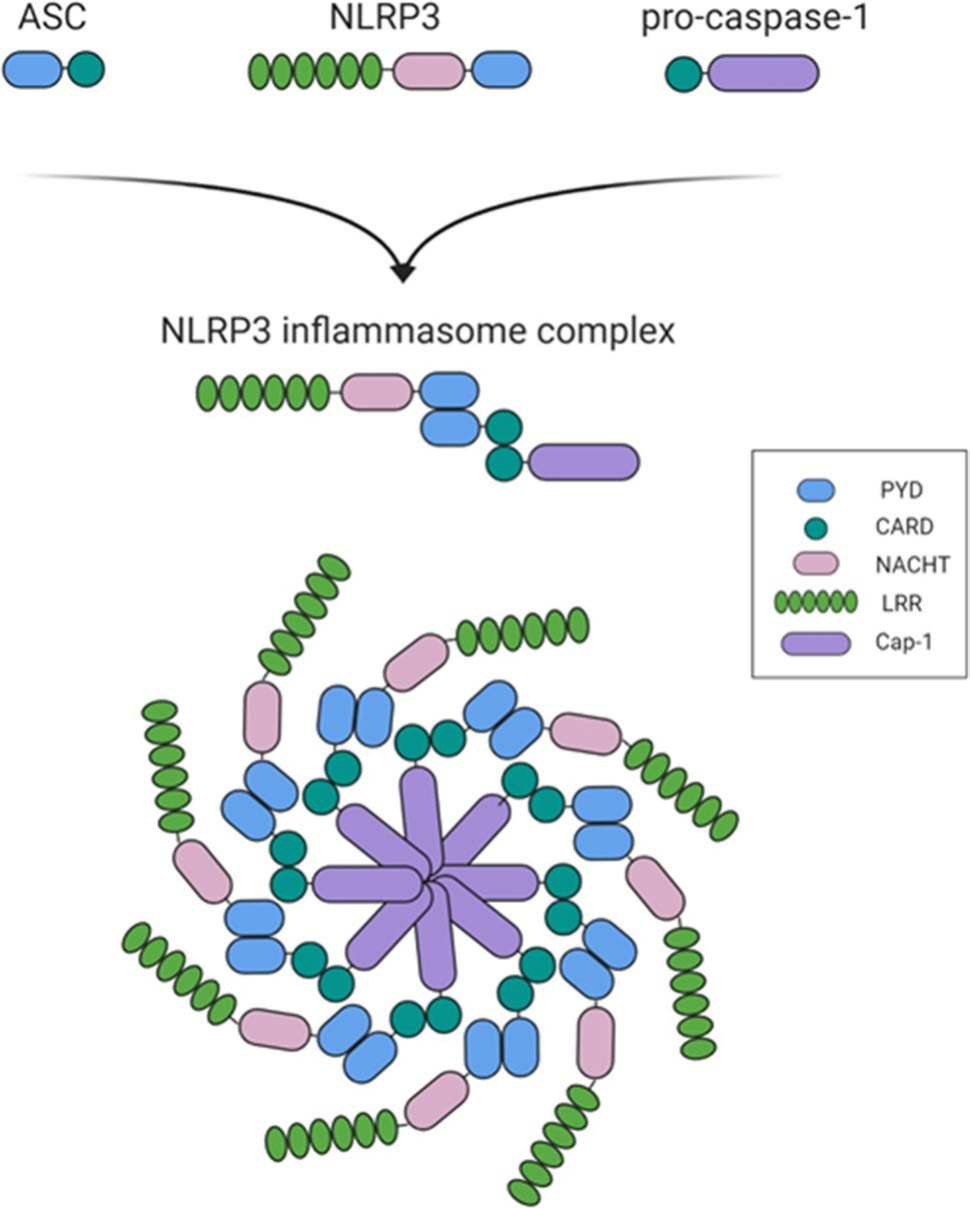
NLRP3 inflammasome structure. The NLRP3 inflammasome is a complex consisting of NLRP3, ASC, and procaspase-1. NLRP3 consists of three regions: the pyrin domain (PYD) in the amino terminus, the NACHT domain, and the leucine-rich repeat domain (LRR) in the carboxy terminus. NLRP3 recruits ASCs through PYD-PYD interactions. In turn, procaspase-1 is recruited by ASC through CARD-CARD interactions to form the NLRP3-ASC-procaspase-1 inflammasome

Once primed, NLRP3 responds to the stimuli and thereby initiates the activation and formation of the inflammasome complex. The stimuli that activate the NLRP3 inflammasome range from pathogen infection and endogenous danger signals to environmental irritants. NLRP3 is a cytosolic receptor composed of three parts: a pyrin domain (PYD) in the amino terminal, a NACHT (nucleotide-binding and oligomerization) domain, and a leucine-rich repeat domain (LRR) in the carboxy terminus. Upon activation, the NACHT domain acquires the ATPase activity required for NLRP3 oligomerization (Abderrazak et al. 2015). Although the LRR domain has been thought to be involved in the recognition of the stimuli (Franchi et al. 2009), its function remains unclear because a recent study showed that the LRR domain was not required for NLRP3 inflammasome activation or NLRP3 self-regulation (Hafner-Bratkovic et al. 2018). The stimuli that activate the NLRP3 inflammasome promote the association of NLRP3, ASC, and procaspase-1 to form the inflammasome complex through PYD-PYD interactions between NLRP3 and ASC and CARD-CARD interactions between ASC and procaspase-1 (Fig. 1).

Pathogens that can activate the NLRP3 inflammasome include yeast (e.g., *Candida albicans* and *Saccharomyces cerevisiae*) (Lamkanfi et al. 2009a; Kumar et al. 2009), bacteria that produce pore-forming toxins (e.g., *Listeria monocytogenes* and *Staphylococcus aureus*) (Gurcel et al. 2006; Sha et al. 2014), and viruses (e.g., Sendai virus, adenovirus, and influenza virus) (Subramanian et al. 2013; Park et al. 2013; Franchi et al. 2014). Endogenous danger signals that induce activation of the NLRP3 inflammasome include ATP, particulates, and oxidized lipids. Uric acid crystals, calcium pyrophosphate dihydrate crystals, fibrillar amyloid-β, and malarial hemozoin are particulate substances that activate the NLRP3 inflammasome and etiological factors in NLRP3 inflammasome-mediated diseases. Oxidized phospholipids are produced from damaged cell membranes and under oxidative stress conditions. 1-Palmitoyl-2-(5-oxovaleroyl)-*sn*-glycero-phosphocholine (POVPC), an oxidized phosphatidylcholine, induces the cleavage of procaspase-1 to caspase-1 (p10) and the cleavage of pro-IL-1β to IL-1β in wild-type mouse macrophages but not in NLRP3-deficient or caspase-1-deficient mouse macrophages (Yeon et al. 2017). In air pouch inflammation and peritonitis mouse models, POVPC injection resulted in the production of caspase-1 (p10), IL-1β, and IL-18 in wild-type mice but not in NLRP3-deficient mice (Yeon et al. 2017). Additionally, environmental irritants, such as silica, asbestos, UV-B irradiation, and skin irritants, induce activation of NLRP3 inflammation (Hornung et al. 2008; Baron et al. 2015; Tavera Busso et al. 2020).

These results show the important role of the NLRP3 inflammasome in protecting the host from exogenous infectious and environmental threats and from endogenous dangers and stress thus contributing to immunity and tissue repair.

## Upstream and intracellular events that activate the NLRP3 inflammasome

**Fig. 2:**
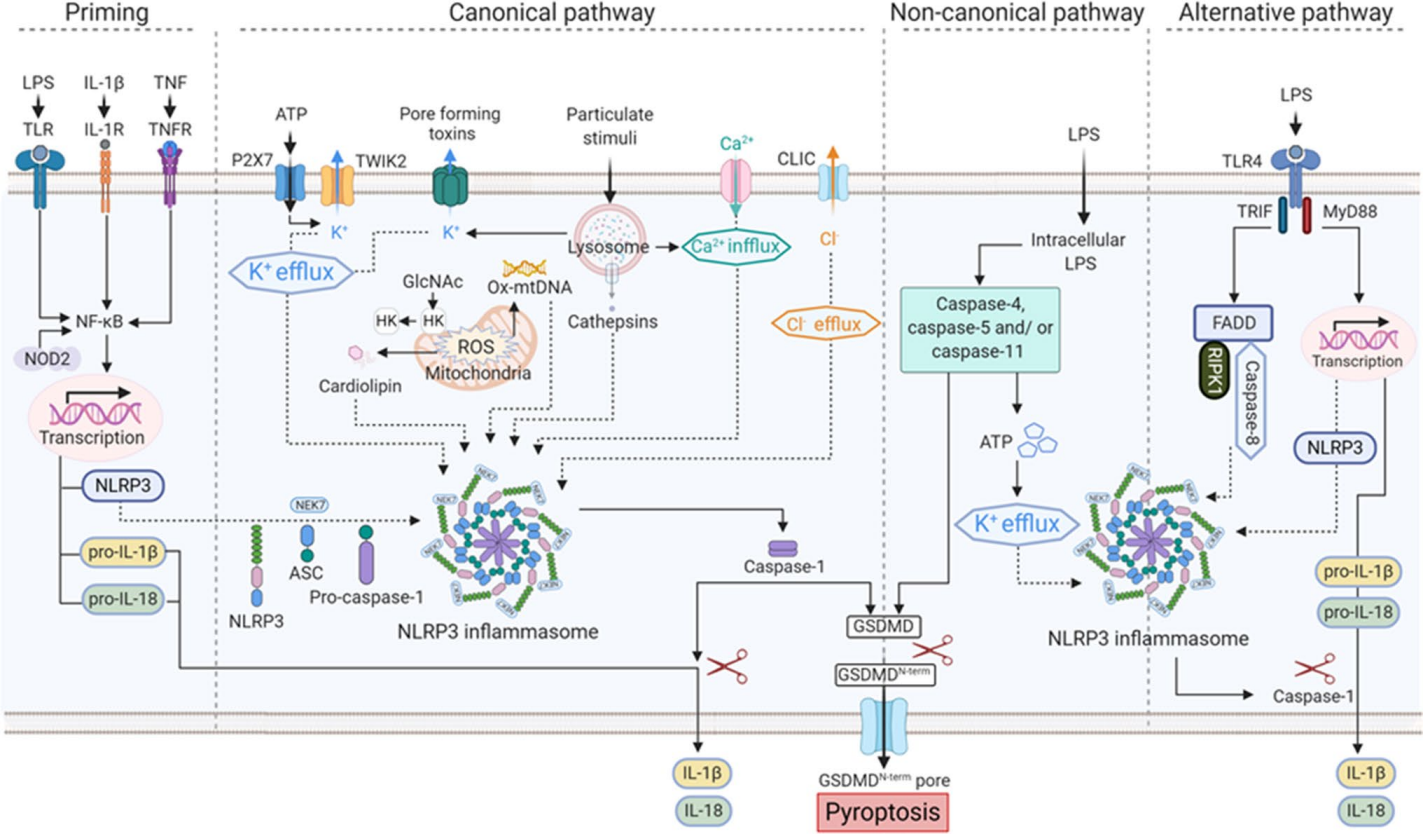
Schematic illustration of the NLRP3 inflammasome pathway. Priming is the first step of NLRP3 inflammasome activation. Priming is induced by Toll-like receptors (TLRs) and nucleotide-binding oligomerization domain-containing protein-2 (NOD2). Priming can also be induced by cytokines, such as tumor necrosis factor (TNF) and interleukin 1 beta (IL-1β), which activate nuclear factor-κB (NF-κB) and gene transcription. Priming upregulates the expression of inflammasome components, such as NLRP3, caspase-1, and pro-IL-1β. The second step is an activation signal mediated by numerous PAMPs, DAMPs, fungi, bacteria producing pore-forming toxins, viruses, and environmental irritants. These activation signals include the efflux of potassium (K^+^) and chloride ions (Cl^−^), the influx of calcium ions (Ca^2+^), lysosomal disruption, mitochondrial dysfunction, metabolic changes, and trans-Golgi disassembly. Decomposition of the bacterial cell wall component peptidoglycan during bacterial infection releases N-acetylglucosamine (GlcNAc) in the lysosomes. Hexokinase (HK), which is located on the mitochondrial membrane, binds GlcNAc. GlcNAc-induced hexokinase relocalization promotes NLRP3 inflammasome activation regardless of K^+^ efflux. Mitochondrial dysfunction and the release of mtROS and mitochondrial DNA (mtDNA) into the cytosol are the major upstream events in NLRP3 activation. NEK7, a regulator of the NLRP3 inflammasome, interacts with NLRP3 through the catalytic domain of NEK7 and the LRR domain of NLRP3. Activation of the inflammasome causes caspase-1 activation resulting in the maturation and release of IL-1β/IL-18 and pyroptosis. Caspase-1 cleaves and releases gasdermin D (GSDMD). The amino terminal cell death domain of GSDMD (GSDMD^Nterm^) inserts into the membrane to form the pores and induce pyroptosis. Noncanonical NLRP3 inflammasome activation is induced by gram-negative bacteria. Intracellular LPS delivered to the cytosol via infection activates human caspase-4/-5 and mouse caspase-11. Human caspase-4/-5 and mouse caspase-11 indirectly induce the activation of pro-IL-1β or pro-IL-18 by promoting NLRP3 inflammasome activation. Activated caspase-4, -5, and -11 cleave GSDMD and cause pyroptosis. The alternative inflammasome pathway is induced by TRIF-RIPK1-FADD-caspase-8 signaling that does not require K^+^ efflux and does not induce pyroptosis

NLRP3 activation includes multiple upstream signals, such as the efflux of potassium (K^+^) and chloride ions (Cl^−^), influx of calcium ions (Ca^2+^), lysosomal disruption, and mitochondrial dysfunction (Fig. 2).

K^+^ efflux is a general requirement for the activation of the NLRP3 inflammasome. ATP-induced activation of the NLRP3 inflammasome is mediated by P2X purinoceptor 7 (P2X7) activation resulting in K^+^ efflux. P2X7 is a ligand-gated ion channel belonging to the purinergic receptor family (Ferrari et al. 2006). Following stimulation by ATP, P2X7 promotes the influx of Ca^2+^ and Na^+^ ions in coordination with the two-pore domain weak inwardly rectifying K^+^ channel 2 (TWIK2), which mediates K^+^ efflux. Nigericin, a microbial toxin derived from *Streptomyces hygroscopicus*, is another well-known NLRP3 inflammasome activator. Nigericin induces K^+^ efflux by acting as a potassium ionophore in a pannexin-1-dependent manner. Particulates, such as alum, silica, and calcium pyrophosphate crystals, cause K^+^ efflux, which is essential for the activation of the NLRP3 inflammasome. In bone marrow-derived macrophages and human monocytes, a low extracellular K^+^ concentration is sufficient to activate the NLRP3 inflammasome. In contrast, a high extracellular K^+^ concentration prevents NLRP3 inflammasome activation (Munoz-Planillo et al. 2013). Therefore, K^+^ efflux is a common upstream signal in most pathways that leads to NLRP3 activation.

The Cl^−^ efflux downstream of K^+^ efflux promotes ASC oligomerization during the formation of the NLRP3 inflammasome (Green et al. 2018). Low extracellular Cl^−^ levels cause ATP-induced IL-1β secretion, whereas Cl^−^ channel blockers and high extracellular Cl^−^ conditions inhibit NLRP3 activation. Chloride intracellular channel proteins (CLICs) are necessary for NLRP3 activation by various stimuli; however, these proteins are not required for the activation of the AIM2 or NLRC4 inflammasome. CLIC1, DLIC4, and DLIC5 deficiency prevents IL-1β efflux (Tang et al. 2017).

Ca^2+^ mobilization is suggested to be another significant upstream event in NLRP3 activation (Murakami et al. 2012). K^+^ efflux controls Ca^2+^ influx since K^+^ acts as the counterion for Ca^2+^ as it enters the cytosol through the plasma membrane (Yaron et al. 2015). Activation of the NLRP3 inflammasome induced by nigericin, alum, and monosodium urate crystals is dependent on Ca^2+^ influx in addition to K^+^ efflux (Murakami et al. 2012; Triantafilou et al. 2013). POVPC‐induced activation of the NLRP3 inflammasome is also mediated through intracellular Ca^2+^ signaling (Yeon et al. 2017). Notably, contradictory evidence suggested that Ca^2+^ influx is not significantly implicated in NLRP3 activation by two canonical NLRP3 agonists, ATP and nigericin (Katsnelson et al. 2015).

Lysosomal rupture is a major event associated with particulates that activates the NLRP3 inflammasome. Phagocytosis of self-derived particulates (e.g., uric acid and cholesterol crystals) and foreign-derived particulates (e.g., alum, silica, and asbestos) is characterized by lysosomal rupture, which leads to the release of the particulates into the cytoplasm. Experiments using cathepsin inhibitors demonstrated that cathepsins residing in the lysosomes are essential for NLRP3 activation by particulate stimuli. Lysosomal rupture by the lysosomotropic dipeptide Leu-Leu-OMe and NLRP3-activating particulate stimuli activate K^+^ efflux and Ca^2+^ influx indicating that these NLRP3 activation pathways converge upstream at K^+^ efflux or Ca^2+^ influx (Katsnelson et al. 2016).

Mitochondrial dysfunction is another major upstream event in NLRP3 inflammasome activation (Zhou et al. 2011). Upon cellular stress, mitochondrial dysfunction leads to excessive production of reactive oxygen species (ROS) and oxidized mitochondrial DNA and the release of these factors into the cytosol (Cruz et al. 2007). Oxidized mitochondrial DNA activates the NLRP3 inflammasome linking mitochondrial apoptotic signaling and inflammasome activation (Shimada et al. 2012). Mitophagy serves as an essential controller of NLRP3 activation since it eliminates damaged and dysfunctional mitochondria and reduces mitochondrial ROS. Treatment with mitophagy inhibitors increases the number of damaged and dysfunctional mitochondria thereby promoting NLRP3 inflammasome activation.

The intracellular events activating the NLRP3 inflammasome are not mutually exclusive and are rather interconnected influencing each other to attain maximal activation of the NLRP3 inflammasome. Additionally, NLRP3 agonists generally share intracellular events leading to NLRP3 inflammasome activation. Considering the in vivo situation when the host is simultaneously exposed to multiple PAMPs and DAMPs, the intracellular signaling pathways of NLRP3 inflammasome activation can be more complex and remain to be defined in detail.

## NLRP3 inflammasome-associated diseases

One of the critical features of the NLRP3 inflammasome is the ability to sense a wide array of danger signals derived from infection, the environment, and the host itself. NLRP3 inflammasome activation is accompanied by inflammatory processes induced by secretion of potent inflammatory cytokines, IL-1β and IL-18, and pyroptotic cell death, which regulate the immune response. However, excessive and persistent activation of the NLRP3 inflammasome is closely linked to pathophysiology of a variety of chronic diseases (Table 1).

**Table 1:** NLRP3 inflammasome-associated diseases

Diseases	Relevance with the NLRP3 inflammasome	References
*Alzheimer's disease*	NLRP3 inflammasome activation by amyloid-β	(Halle et al. 2008)
*Autoimmune diseases*		
Multiple sclerosis (MS) Rheumatoid arthritis (RA) Systemic lupus erythematosus (SLE)	NLRP3 gene SNP and increase of NLRP3, caspase-1, ASC, IL-1β, and IL-18 in MS patients Increased expression of NLRP3 inflammasome components in RA patients Controversial in a mouse model and SLE patients	(Keane et al. 2018) (Kastbom et al. 2008) (Tsai et al. 2011)
*Autoinflammatory diseases*		
Cryopyrin-Associated Periodic Syndromes (CAPS) Familial cold autoinflammatory syndrome (FCAS) Muckle-Wells syndrome (MWS) Neonatal Onset Multisystemic Inflammatory Disease (NOMID)/Chronic Infantile Neurologic Cutaneous Articular (CINCA)	Heterozygous gain-of-function mutations within the NLRP3 gene Increase of NLRP3, IL-1β, and IL-18 in CAPS patients	(Masters et al. 2009) (Morandini et al. 2014) (Booshehri and Hoffman 2019)
*Cancer*	Different effects on tumor formation, development and invasion depending on the type and stage of cancer	(El-Omar et al. 2000) (Snoussi et al. 2005) (Zaki et al. 2010) (Chen et al. 2013) (Bae et al. 2017)
*Cardiovascular diseases*		
Atherosclerosis Acute myocardial infarction	Activation and inhibition study NLRP3 inflammasome activation by cholesterol crystals	(Sandanger et al. 2013) (Duewell et al. 2010)
*Gout*	NLRP3 inflammasome activation by uric acid crystals	(Landis and Haskard 2001)
*Nonalcoholic fatty liver disease (NAFLD)*	Involvement in NASH/NAFLD patients and mouse models	(Dixon et al. 2012)
*Silicosis*	NLRP3 inflammasome activation by silica	(Hornung et al. 2008)
*Skin diseases*		
Acne Atopic dermatitis (AD) Psoriasis Vitiligo	NLRP3 and caspase-1 activation by P. acnes in sebocytes Lowered NLRP3 and caspase-1 in lesional AD skin NLRP3 gene SNP in psoriatic lesions NLRP3 inflammasome activation by monobenzone in melanocytes	(Li et al. 2014) (Dai et al. 2011) (Carlstrom et al. 2012) (van den Boorn et al. 2016)
*Type 2 diabetes (T2D)*	The relevance of NLRP3, ASC, caspase-1 or IL-1β in T2D Studied in a high fat diet-induced obesity mouse model	(Vandanmagsar et al. 2011)

### Alzheimer's disease

Accumulation of amyloid-β plaques in the cerebrum is the main characteristic of Alzheimer’s disease (Masters and Selkoe 2012). The NLRP3 inflammasome in microglia acts as an amyloid-β sensor after phagocytosis of amyloid-β accompanied by lysosomal damage and release of cathepsin B eventually culminating in IL-1β secretion (Halle et al. 2008; Sebastian-Valverde and Pasinetti 2020). The levels of cleaved caspase-1 were increased in the brain of patients with Alzheimer’s disease and mild cognitive impairment (Heneka et al. 2013). Similarly, an increase in caspase-1 cleavage and IL-1β levels was observed in the brain of aged APP/PS1 transgenic mice (Heneka et al. 2013). Deficiency of NLRP3 and caspase-1 resulted in protection from cognitive impairment and memory loss in an APP/PS1 mouse model of Alzheimer's disease (Heneka et al. 2013). Additionally, NLRP3 deficiency led to a decrease in the amyloid-β levels and deposition suggesting an aggravating role of the NLRP3 inflammasome in the pathology of Alzheimer’s disease (Heneka et al. 2013).

In a mouse model of sporadic Alzheimer’s disease (SAD) induced by streptozotocin (STZ) injection, the levels of NLRP3 in the cortex and hippocampus were increased in the STZ group compared with those in the sham group (He et al. 2020). The symptoms of SAD, such as neuronal dysfunction, neuronal loss, and amyloid-β deposition, were aggravated by LPS treatment in an NLRP3-dependent manner, and genetic deletion of NLRP3 or an NLRP3 inhibitor, MCC950, prevented SAD symptoms via inhibition of NLRP3 inflammasome activation (He et al. 2020).

Administration of OLT1177, an NLRP3 inflammasome inhibitor, rescued cognitive impairment and neuroinflammation in an APP/PS1 mouse model of Alzheimer’s disease (Lonnemann et al. 2020). Activation of the NLRP3 inflammasome was increased in CRND8 APP transgenic mice, an Alzheimer's disease mouse model, compared to nontransgenic littermate controls, and treatment with JC-124, another NLRP3 inflammasome inhibitor, led to a decrease in amyloid-β deposition in the brain of CRND8 transgenic mice (Yin et al. 2018).

Aggregation of hyperphosphorylated tau is another important etiology of neurodegeneration in addition to accumulation of amyloid-β in plaques (Lewis and Dickson 2016). Alzheimer's disease is considered to be a secondary tauopathy (Lewis and Dickson 2016). Primary tauopathies, such as frontotemporal dementia (FTD), present neuroinflammation and cognitive impairment. Caspase-1 cleavage, ASC levels, and mature IL-1β were elevated in the cortex samples of FTD patients carrying a tau mutation and in the cerebral samples of 11-month-old Tau22 mice (Lewis and Dickson 2016). Amyloid-β-induced tau pathology was mediated by the NLRP3 inflammasome, and deficiency in NLRP3 and ASC decreased tau pathology and improved cognition in Tau22 mice (Lewis and Dickson 2016).

ASC specks accumulate in the extracellular space after NLRP3 inflammasome activation and act as a danger signal by inducing lysosomal damage, nucleation of soluble ASC in the cytosol, caspase-1 activation, and IL-1β production after uptake by the cell (Franklin et al. 2014). ASC specks function as a seed to induce aggregation of soluble ASC to form fibrillar structures similar to other prionoid proteins (Franklin et al. 2014). Similarly, amyloid-β is aggregated into large and insoluble amyloid fibrils, which assemble into amyloid plaques (Chen et al. 2017). ASC specks released by microglia enhanced amyloid-β aggregation, and the binding of ASC specks to amyloid-β was increased in the brain of Alzheimer’s disease patients (Venegas et al. 2017).

Overall, activation of the NLRP3 inflammasome aggravates the pathologies of Alzheimer's disease by inducing inflammation and tissue damage. Pharmacological inhibitors of NLRP3 can be a beneficial strategy for the treatment of Alzheimer's disease.

### Autoimmune diseases

#### Multiple sclerosis

Multiple sclerosis (MS) is a chronic inflammatory autoimmune disease of the central nervous system. Hereditary susceptibility is one of the leading causes of MS pathology. The single-nucleotide polymorphism (SNP) rs35829419 in the NLRP3 gene was found to be associated with MS severity (Soares et al. 2019). Moreover, a recent study detected mutations in the NLRP3 and caspase-1 genes in MS patients (Vidmar et al. 2019). Elevated IL-18 has been reported to be associated with the clinical progression of MS (Losy and Niezgoda 2001). In addition, the NLRP3 inflammasome was found to be overactive in monocytes from MS patients (Malhotra et al. 2020). An increase in caspase-1, NLRP1, NLRP3, and AIM2 expression and IL-1β production was detected in the white matter of MS patients (Voet et al. 2019). High protein levels of caspase-1, ASC, and IL-18 were detected in the serum of MS patients (Keane et al. 2018). These results suggest that the NLRP3 inflammasome may be a promising prognostic factor and therapeutic target.

#### Rheumatoid arthritis

NLRP3/CARD8^−/−^ genetic mutations are common in patients with rheumatoid arthritis and are associated with an increase in incidence and severity of rheumatoid arthritis symptoms (Kastbom et al. 2008). Conversely, transcription of the genes of the components of the NLRP3 inflammasome was substantially increased in patients with rheumatoid arthritis (Mathews et al. 2014). In caspase-1^−/−^ mice, the symptoms of rheumatoid arthritis, including joint inflammation and cartilage degeneration, were significantly reduced (Joosten et al. 2009).

#### Systemic lupus erythematosus (SLE)

In patients with SLE, the NLRP3 inflammasome was reported to be overactivated (Kahlenberg and Kaplan 2014). NLRP3 gene expression was significantly increased concomitant to the progression of lupus nephritis in an MRL/lpr lupus model. The NLRP3 inflammasome and caspase-1 contribute to severe organ damage and are essential for autoantibody production and nephritis development (Lu et al. 2017). Controversial reports showed that NLRP3 downregulation is correlated with disease incidence in SLE patients (Yang et al. 2014). Activation of the NLRP3 inflammasome resulted in a decrease in lupus development in mice (Tsai et al. 2011). Specific role of the NLRP3 inflammasome in SLE remains to be fully elucidated.

### Autoinflammatory diseases

Mutations in NLRP3 are a well-known etiology of a series of autoinflammatory diseases, such as cryopyrin-associated periodic syndrome (CAPS). CAPS is a rare hereditary autoinflammatory disease with fever and continuous systemic inflammation (Mensa-Vilaro et al. 2016). CAPS includes a continuum of three phenotypes: familial cold autoinflammatory syndrome (FCAS), Muckle-Wells syndrome (MWS), and neonatal-onset multiple systems inflammatory disease (NOMID)/chronic infantile neurologic cutaneous articular (CINCA) syndrome. These CAPS phenotypes were previously thought to be separate conditions; however, currently, these phenotypes are used to describe the severity of the disease. Chronicity or severity of shared symptoms is most severe in CINCA/NOMID followed by MWS and FCAS. FCAS is characterized by recurrent urticaria and fever after common exposure to cold (Stych and Dobrovolny 2008). MWS is an intermediate phenotype initially described by Muckle and Wells in 1962 (Muckle and Wellsm 1962), who determined that the condition was inherited in an autosomal dominant manner. The most severe form is NOMID, which was described for the first time in 1973. Development of neonatal skin symptoms with damage to the terminal organs is the hallmark of NOMID. These symptoms include arthrosis, chronic urticaria, and symptoms related to the central nervous system (CNS). In these diseases, inflammation can occur spontaneously without obvious irritation or in response to triggers, such as cold, stress, or exercise.

In patients with CAPS, single amino acid mutations in NLRP3 cause the hypersecretion of IL-1β and severe inflammatory symptoms (Booshehri and Hoffman 2019). Approximately 100 pathogenic mutations are known to be associated with CAPS. Most CAPS-related mutations are located in exon 3, which encodes the NOD domain, and several mutations in the C-terminal exons encoding the LRR domain have been reported (Masters et al. 2009). A strong genotype–phenotype correlation along the disease continuum has been reported. CAPS monocytes secrete excessive amounts of ATP resulting in a high level of NLRP3 inflammasome activation and the production of IL-1β and IL-18 (Morandini et al. 2014). In CAPS, excessive production and secretion of IL-1β are the key elements implicated in the development of chronic inflammation.

### Cancer

Inflammation induced by microbial or danger signals affects all stages of tumor development, and proinflammatory cytokines, including IL-1β and IL-6, are important mediators of inflammation-induced tumorigenesis (El-Omar et al. 2000). Increasing attention is focused on identification of the role of the NLRP3 inflammasome in various tumor types, while the role of NLRP3 inflammasome activation in tumor formation, development, and invasion remains controversial depending on types and stages of cancer (Moossavi et al. 2018). Studies on colitis-associated colorectal cancer suggested that NLRP3 plays a protective role in inflammatory components against carcinogenesis mediated by the antitumor effect of IL-18 (Zaki et al. 2010). In contrast, in lung cancer (Wang et al. 2016), prostate cancer (Chen et al. 2013), breast cancer (Weichand et al. 2017), and head and neck squamous cell carcinoma (Bae et al. 2017), the NLRP3 inflammasome, IL-1β, and IL-18 promote tumor growth, proliferation, invasion, and metastasis. In addition, the NLRP3 inflammasome is associated with chemo- and radioresistance in glioblastoma and oral squamous cell carcinoma. The role of the NLRP3 inflammasome in the tumor microenvironment has been noted. Wild-type macrophages stimulated with ATP enhanced the metastatic potential of melanoma cells; however, NLRP3- or caspase-1-deficient macrophages lost the ability to promote metastasis of melanoma cells (Lee et al. 2019a). IL-1β may participate in NLRP3 inflammasome-mediated tumor promotion in the tumor microenvironment since recombinant IL-1β treatment of macrophages increased the migration and invasion of melanoma cancer cells (Lee et al. 2019a). Further research is required to determine the potential therapeutic role of the NLRP3 inflammasome in human malignancies.

### Cardiovascular diseases

#### Acute myocardial infarction

NLRP3 is the most extensively characterized inflammatory sensor in the heart and is activated in response to noninfectious stimuli, such as cell debris or cholesterol, which act as DAMPs (Liu et al. 2013a). During acute myocardial infarction, injury results from a decrease in blood supply to the tissue, which is worsened by an inflammatory response stimulated during reperfusion (Westman et al. 2016). Activation of the NLRP3 inflammasome triggers myocardial damage through the release of IL-1β and promotion of inflammatory cell death by pyroptosis (Takahashi 2014). Inhibition of NLRP3 inflammasome activation during the initial reperfusion period reduces the overall infarct size and preserves normal cardiac function in animal models of myocardial ischemia–reperfusion injury (Sandanger et al. 2013). IL-1 blockade can prevent recurrence of acute myocardial infarction in patients who have experienced previous events (Van Tassell et al. 2017). IL-1 blockade can prevent heart failure and improve motor skills and heart function in heart failure patients (Ridker et al. 2011).

#### Atherosclerosis

Atherosclerosis is a chronic inflammatory disease in which imbalanced lipid metabolism causes a gradual narrowing of arterial blood vessels. Cholesterol crystals produced intracellularly activate the NLRP3 inflammasome in mouse and human cells via phagolysosomal damage that depends on cathepsin B and cathepsin L (Duewell et al. 2010; Altaf et al. 2015). IL-1β and IL-18 are produced in response to inflammasome activation and play essential roles in the initiation and progression of atherosclerosis. Deficiencies in IL-1β and IL-18 reduced the lesion size in an apolipoprotein E-deficient mouse model of atherosclerosis (Zhang et al. 1992).

### Gout

Gout is a chronic disease manifested as deposition of monosodium urate (MSU) crystals that are formed under increased urate concentrations (Brill and McCarty 1964). MSU crystals are well-known activators of the NLRP3 inflammasome. NLRP3 inflammasome activation and the release of IL-1β have essential roles in gout by triggering acute gout flares (Landis and Haskard 2001). In NLRP3 inflammasome-deficient mice, the symptoms of gouty arthritis and pathological markers induced by MSU injection were lessened compared to those of the wild-type control group (Martinon et al. 2006).

### Nonalcoholic fatty liver disease (NAFLD)

NAFLD is a broad-spectrum liver disease ranging from simple steatosis to liver failure (Tiniakos et al. 2010). Excessive accumulation of hepatic lipids has been proposed to be the primary etiological factor of NAFLD to accelerate the progression to nonalcoholic steatohepatitis (NASH) (Tilg and Moschen 2010). Hepatic NLRP3 inflammasome activation was observed in NASH patients because pro-IL-1β and pro-IL-18 were markedly increased in the liver of NASH patients. These findings may lead to new treatment strategies to prevent hepatic steatosis from progressing to a more serious form of the disease (Wree et al. 2014). Studies of knockout mice deficient in NLRP3 inflammasome components, including NLRP3, ASC, and caspase-1, showed that NLRP3 inflammasome activation was essential for NAFLD progression in an MCD-induced NAFLD model (Dixon et al. 2012). Caspase-1-knockout mice fed a high-fat diet showed improved disease status associated with obesity and insulin resistance compared to that in high-fat diet-fed wild-type mice (Stienstra et al. 2010).

### Silicosis

The pathophysiology of silicosis includes deposition of silica particles in pulmonary alveoli accompanied by silica-mediated activation of the NLRP3 inflammasome in macrophages (Hornung et al. 2008). Inhalation of silica crystals causes inflammation of the alveolar space, and long-term exposure to silica can lead to silicosis, a fibrotic lung disease. In addition, deposition of macrophage receptors with collagenous structures (MARCO), NLRP3, caspase-1, ASC, IL-1β, and IL-18 was found in both glomerular and tubulointerstitial areas of patients with a history of silicosis (Chen et al. 2019).

### Skin diseases

#### Acne

*Propionibacterium acnes* (*P. acnes*) induces the expression of proinflammatory cytokines in a variety of cells involved in skin innate immunity (Graham et al. 2004). IL-1β expression is upregulated in the sebaceous glands of acne lesions. Stimulation of human myeloid cells with *P. acnes* significantly enhanced caspase-1 activation and IL-1β secretion (Kistowska et al. 2014). Moreover, knocking down the expression of NLRP3 abolished *P. acnes*-induced IL-1β production in sebaceous cells. The activation of the NLRP3 inflammasome by *P. acnes* was dependent on protease activity and generation of reactive oxygen species. In addition, NLRP3-deficient mice showed impaired inflammatory responses to *P. acnes* (Li et al. 2014). These results suggest that human sebaceous cells are important immunocompetent cells that induce NLRP3 inflammasome activation and that IL-1β activation induced by *P. acnes* in the sebaceous glands may play a role in acne pathogenesis.

#### Atopic dermatitis

Atopic dermatitis is a chronic inflammatory disease caused by a combination of genetic and environmental factors. *Dermatophagoides pteronyssinus*, a house dust mite allergen, is an important etiology for the development of atopic dermatitis and induces assembly of the NLRP3 inflammasome complex resulting in caspase-1 activation and IL-1β and IL-18 release from keratinocytes (Dai et al. 2011). This effect indicates a positive role of the NLRP3 inflammasome in house dust mite-induced atopic dermatitis (Dai et al. 2011).

Th2 responses are dominant in the acute phase of atopic dermatitis, and the appearance of high levels of IFN-γ and Th1 cells is observed in the chronic phase (Grewe et al. 1995). The expression of NLRP3 and caspase-1 was lower in atopic dermatitis skin lesions compared to those in healthy skin (Niebuhr et al. 2014). Th2 cytokines, such as IL-4, IL-5, and IL-13, reduced the expression of NLRP3 and ASC, whereas a Th1 cytokine, interferon-γ, increased the expression of NLRP3 in primary keratinocytes (Niebuhr et al. 2014). The exotoxin α-toxin from *Staphylococcus aureus* (*S. aureus*) induced IL-1β secretion mediated by caspase-1 activation in monocytes from healthy controls, and IL-1β secretion by α-toxin was impaired in monocytes from atopic dermatitis patients (Niebuhr et al. 2014). These results indicate that impairment of the NLRP3 inflammasome leads to increased vulnerability to *S. aureus*-mediated skin inflammation in atopic dermatitis patients (Niebuhr et al. 2014).

Single-nucleotide polymorphisms (SNPs) of the *Nlrp3* gene are associated with atopic dermatitis. There is a strong association between *NLRP3* variant rs10733113 and an increase in the levels of serum IgE-specific antibodies in male individuals of Swedish families with atopic dermatitis (Bivik et al. 2013). A significant correlation between the NLRP3 rs35829419 polymorphism and increased susceptibility to atopic dermatitis has been identified (Zhang et al. 2015).

NLRP3 inflammasome plays a positive role in the development of atopic dermatitis by house dust mite allergens, while impaired NLRP3 inflammasome activity under Th2-skewed conditions makes atopic dermatitis patients susceptible to *S. aureus*-mediated skin infection. Therefore, the NLRP3 inflammasome is apparently involved in the pathology of atopic dermatitis depending on the context of whether its activation has detrimental or beneficial effects on the manifestations of the disease.

#### Psoriasis

Psoriatic skin is characterized by hyperproliferation and abnormal differentiation of keratinocytes and infiltration of inflammatory cells. The association between inflammation and psoriasis has been investigated due to the relationship with inflammatory cytokines, such as IL-1β and IL-18. Studies have shown that polymorphisms in NLRP1, NLRP3, and CARD8 are associated with susceptibility to psoriasis (Carlstrom et al. 2012; Ekman et al. 2014). The activity of caspase-1 was higher in psoriatic lesions than that in nonlesional skin (Johansen et al. 2007).

#### Vitiligo

Vitiligo is an autoimmune skin disease that leads to bleaching and white spots due to the loss of functioning melanocytes (Ezzedine et al. 2015). Skin exposed to monobenzone displays melanocyte-specific inflammation characterized by macrophage infiltration and activation of NK cells. Melanocyte-specific immune response was significantly suppressed in NLRP3-deficient mice suggesting that the NLRP3 inflammasome is the key to monobenzone-induced inflammation in melanocytes (van den Boorn et al. 2016). In addition, inactivation of the NLRP3 inflammasome in keratinocytes impaired CD8^+^ T cell recruitment and inhibited cutaneous T-cell response in patients with vitiligo. This result indicates that NLRP3 inflammasome activation and its downstream cytokines may be promising therapeutic targets for the treatment of patients with vitiligo (Li et al. 2020).

### Type 2 diabetes (T2D)

T2D is a chronic inflammatory disease and a global threat caused by insulin resistance (Donath and Shoelson 2011). IL-1β is closely related to the development of T2D by promoting insulin resistance, damaging β-cell functions, and causing cell death. Treatment with an IL-1 receptor antagonist (IL-1RA) or anti-IL-1 antibody improves blood glucose homeostasis and β-cell functions (Larsen et al. 2007). The levels of NLRP3, ASC, and proinflammatory cytokines were increased in monocyte-derived macrophages isolated from T2D patients. The upregulation of interleukin (IL)-1β maturation, IL-18 secretion, and caspase-1 cleavage in response to various NLRP3 activators was observed in monocyte-derived macrophages from T2D patients (Lee et al. 2013). Furthermore, mice deficient in NLRP3, ASC, or caspase-1 showed improved glucose metabolism and insulin sensitivity when exposed to a high-fat diet (Vandanmagsar et al. 2011). This effect was mediated by the normalization of the insulin-PI3K-Akt signaling pathway and a decrease in the concentrations of proinflammatory cytokines in the context of NLRP3, ASC, or caspase-1 deficiency (Vandanmagsar et al. 2011).

Saturated fatty acids (FAs), such as palmitate, and ceramide are increased in subjects fed a high-fat diet and can cause T2D at least partly mediated by NLRP3 inflammasome activation. Palmitate induces mitochondrial ROS generation, which activates the NLRP3 inflammasome (Wen et al. 2011). Ceramide induces NLRP3-dependent caspase-1 activation in macrophages and adipose tissue (Vandanmagsar et al. 2011). In contrast, omega-3 unsaturated fatty acids suppressed NLRP3 inflammasome activation in a high-fat diet-induced T2D model (Yan et al. 2013).

Thus, accumulating evidence demonstrates the significant role of the NLRP3 inflammasome in the development and progression of chronic diseases. Many chronic diseases can be initiated or aggravated by signals activating the NLRP3 inflammasome, including DAMPs derived from tissue and cell damage and PAMPs derived from infections. These considerations emphasize the importance of controlling the NLRP3 inflammasome to maintain danger surveillance and proper immune responses.

## Therapeutic targeting of the NLRP3 inflammasome

Continuing studies on NLRP3 result in the rapidly progress in the development of therapeutic strategies targeting NLRP3 in many diseases. Current treatments for NLRP3-related pathologies involve suppression of the activation of the NLRP3 inflammasome by direct regulation of the component of the NLRP3 inflammasome or indirect regulation of associated cellular signaling events (Table 2).

**Table 2:** Inhibitors of the NLRP3 inflammasome

Mechanism	Agents	Targets	Diseases	References
Direct targeting of NLRP3	Bay 11–7082	NLRP3 NACHT domain (ATPase region)	Systemic lupus erythematosus	(Juliana et al. 2010)
	β‐carotene	NLRP3 PYD domain	Gout	(Yang et al. 2020a)
	CY-09	NLRP3 NACHT domain (Walker A motif)	Gout, T2D, CAPS	(Jiang et al. 2017)
	MNS	NLRP3 LRR and NACHT domains	Inflammation-associated diseases	(He et al. 2014)
	MCC950	NLRP3 NACHT domain (Walker B motif)	Multiple sclerosis, CAPS	(Coll et al. 2015)
	OLT1177	NLRP3 NACHT domain (ATPase region)	Degenerative arthritis, CAPS	(Marchetti et al. 2018a)
	Oridonin	NLRP3 NACHT domain (cysteine 279)	T2D, peritonitis, gout	(He et al. 2018)
	Parthenolide	NLRP3 NACHT domain (cysteine modification)	Cystic fibrosis	(D'Anneo et al. 2013)
	Tranilast	NLRP3 NACHT domain	Gout, CAPS, T2D	(Huang et al. 2018)
Direct targeting of ASC	Caffeic acid phenethyl ester	ASC	Gout	(Lee et al. 2016)
Direct targeting of caspase-1	VX-740, VX-765	Caspase-1	Rheumatoid arthritis, epilepsy, psoriasis, AD, myocardial infarction	(Linton 2005)
Targeting IL-1β	Anakinra	IL-1α and IL-1β	Gout, rheumatoid arthritis coronary artery disease, acute Kawasaki disease	(So et al. 2007)
	Canakinumab	IL-1β	CAPS, gout	(Church and McDermott 2010)
	Gevokizumab	IL-1β	T1D, T2D, Rheumatoid arthritis	(Cavelti-Weder et al. 2012)
	Rilonacept	IL-1	CAPS, gout, systemic sclerosis	(Kone-Paut and Galeotti 2015)
Targeting IL-18	GSK1070806	IL-18	Autoimmune diseases, non-Hodgkin’s lymphoma, IBD	(Mistry et al. 2014)
Indirect inhibition	Auranofin	IKK kinase, thioredoxin reductase	Rheumatoid arthritis, acne	(Isakov et al. 2014)
	β-hydroxybutyrate	K + efflux, ASC aggregation	MWS, FCAS, urate crystal–induced peritonitis	(Youm et al. 2015)
	Celastrol	K + efflux	Autoimmune diseases, tumor	(Lee et al. 2019a)
	EGCG	Mitochondrial DNA	Gout	(Lee et al. 2019b)
	FC11A-2	Caspase-1, IL-1β, IL-18	Colitis	(Liu et al. 2013b)
	Glyburide, 16,673–34-0, JC171	ATP-sensitive K + channels, ASC, P2X7 signaling	T2D, acute myocardial infarction	(Lamkanfi et al. 2009b) (Marchetti et al. 2014) (Guo et al. 2017)
	JC124	ASC aggregation	Traumatic brain injury, neuroinflammatory, Acute myocardial infarction, AD	(Yin et al. 2018)
	Licochalcone A	Mitochondrial ROS, UCP1 expression	*P. acnes*-mediated acne, obesity	(Lee et al. 2018)
	Sulforaphane	AMP-activated protein kinase/autophagy axis	NAFLD, gout	(Yang et al. 2016)

### Direct targeting of NLRP3

#### Bay 11-7082

Bay 11-7082, a phenyl vinyl sulfone-related compound, was shown to prevent the organization of the ASC pyroptosome and NLRP3 inflammasome through alkylation of cysteine residues in the ATPase region of NLRP3 (Juliana et al. 2010). Vinyl sulfone derivatives were well tolerated and nonmutagenic and had suitable pharmacokinetic profiles in preclinical trials suggesting feasibility of their application. These compounds have been used as antiparasitic agents in dogs and mice (Kerr et al. 2009). However, Bay 11–7082 inhibits the kinase activity of IKKβ resulting in the modulation of the NF-κB pathway, which is upstream of NLRP3 expression. Thus, the specificity of Bay 11–7082 toward the NLRP3 inflammasome is uncertain.

#### β‐Carotene (provitamin A)

β‐Carotene suppresses NLRP3 inflammasome activation induced by ATP, MSU crystals, and nigericin in macrophages. β‐Carotene binds directly to the pyrin domain (PYD) of NLRP3. In a mouse model of gouty arthritis, inflammatory symptoms caused by MSU crystals were attenuated by oral administration of β‐carotene. In addition, β-carotene reduced IL-1β secretion by human synovial cells isolated from patients with gout showing a potential inhibitory effect of β‐carotene on human gout (Yang et al. 2020a).

#### CY-09

CY-09 is an analog of cystic fibrosis transmembrane conductance regulator (CFTR) channel blockers (Ma et al. 2002). In mouse macrophages primed with LPS, CY-09 blocked ATP-, monosodium urate (MSU)-, and nigericin-induced caspase-1 activation and IL-1β release (Jiang et al. 2017). This inhibitory effect did not depend on posttranslational modification (ubiquitination) of NLRP3. Mechanistically, to remove ATP bound to NLRP3, CY-09 interacts with the NLRP3 Walker A motif directly without affecting NLRP1, NLRC4, RIG-1, or NOD2 (Jiang et al. 2017). CY-09 exerted outstanding preventive or therapeutic effects in mouse models of gout, T2D, and CAPS.

#### 4-Methylenedioxy-β-nitrostyrene (MNS)

MNS binds to the LRR and NACHT domains of NLRP3 (He et al. 2014) and directly targets these domain to block the ATPase activity of NLRP3. MNS does not affect the activation of the AIM2 or NLRC4 inflammasomes (He et al. 2014).

#### MCC950

MCC950, a diarylsulfonylurea-containing compound, was found to be a selective inhibitor of the NLRP3 inflammasome. MCC950 blocks the canonical (e.g., ATP, nigericin, and monosodium urate) and noncanonical (e.g., cytosolic LPS) activation of NLRP3 at nanomolar concentrations and the AIM inflammasome at relatively higher concentrations; however, MCC950 does not affect the NLRP4 inflammasome (Coll et al. 2015). MCC950 reduced IL-1β production and the severity of experimental autoimmune encephalomyelitis (EAE), an animal disease model of multiple sclerosis. MCC950 rescued neonatal lethality in a mouse model of CAPS (Coll et al. 2015). MCC950 has been shown to block the Walker B motif within the NLRP3 NACHT domain (Coll et al. 2019) to block ATP hydrolysis by NLRP3 required for inflammasome functions. Extensive pharmacokinetic investigations in vitro and in vivo resulted in significant progress toward therapeutic applications (Coll et al. 2019).

#### OLT1177

OLT1177 is a β-sulfonyl nitrile compound. OLT1177 can block both canonical and noncanonical activation of the NLRP3 inflammasome. OLT1177 directly binds to NLRP3 to block its ATPase activity and decrease caspase-1 activity and IL-1β secretion in monocytes from CAPS patients and reduced LPS-induced systemic inflammation in mice (Marchetti et al. 2018b). A phase I clinical trial for the treatment of degenerative arthritis was completed, and a phase II clinical trial is ongoing (Toldo and Abbate 2018). In the phase I trial, oral administration of OLT1177 to healthy subjects had an excellent safety profile and level of tolerance. Despite long half-life, the compound did not show any signs of organ or hematological toxicities at various doses (Marchetti et al. 2018a; Sanchez-Fernandez et al. 2019). Therefore, OLT1177 may have great potential for the treatment of NLRP3-related diseases.

#### Oridonin

Oridonin (Ori) is a bioactive ent-kaurane diterpenoid that is a primary component of the herbal plant *Rabdosia rubescens* and is widely used in traditional Chinese medicine (Kadota et al. 1997). Previously, Ori was reported to interact with cysteine 279 of the NLRP3 NACHT domain through a covalent bond and abolish NLRP3-NEK7 interactions resulting in selective inhibition of NLRP3 inflammasome activation. Use of Ori in mouse models of T2D, peritonitis, and gouty arthritis resulted in significant preventive and therapeutic effects (He et al. 2018).

#### Parthenolide

Parthenolide, a plant sesquiterpene lactone with anti-inflammatory properties, is used as an herbal medicine to treat various inflammatory diseases (Heinrich et al. 1998). Parthenolide was originally known to be an NFκB inhibitor acting by inhibiting the kinase activity of κB kinase β (IKKβ). Parthenolide inhibits NLRP1, NLRC4, and NLRP3 stimuli by alkylating a number of cysteine residues of caspase-1 thus blocking caspase-1 activation (Juliana et al. 2010). Additionally, parthenolide may directly target the ATPase activity of NLRP3 through cysteine modification. Parthenolide has poor solubility and bioavailability, and soluble analogs of parthenolide are currently undergoing evaluation (D'Anneo et al. 2013).

#### Tranilast

Tranilast (N-[3′,4′-dimethoxycinnamoyl]-anthranilic acid (TR)) is a tryptophan metabolite analog that has inhibitory effects on homologous passive cutaneous anaphylaxis (Darakhshan and Pour 2015). Inhibitory effects of TR were selective for the NLRP3 inflammasome. TR impaired the endogenous NLRP3-ASC interaction, which was verified by its binding with the NLRP3 NACHT domain and suppression of direct NLRP3-NLRP3 interactions. TR showed significant therapeutic and preventive effects in the mouse models of CAPS and T2D (Huang et al. 2018). TR is a reasonably safe compound; patients showed moderate levels of tolerance to high doses of TR (Platten et al. 2005).

### Direct targeting of ASC

#### Caffeic acid phenethyl ester (CAPE)

CAPE inhibited NLRP3 inflammasome activation by blocking caspase-1 activation and IL-1β production induced by MSU crystals. CAPE directly associates with ASC to block the NLRP3-ASC interaction induced by MSU crystals (Lee et al. 2016). In a mouse gouty arthritis model, oral administration of CAPE inhibited MSU crystal-induced caspase-1 activation and IL-1β production in air pouch exudate and foot tissue thus attenuating inflammatory symptoms (Lee et al. 2016).

### Direct targeting of caspase-1

#### VX-740 and VX-765

VX-740 (pralnacasan) and its analog VX-765 are peptidomimetic inhibitors of caspase-1. VX-765 is a pro-drug of VRT-043198, which is a potent inhibitor of caspase-1. VX-765 reduces IL-1β and IL-18 levels both in vitro and in vivo in correlation with tissue-protective effects in animal models of inflammatory disease. VX-740 and VX-765 act by covalently modifying the catalytic cysteine residues in the active sites of caspase-1 to inhibit enzymatic activity (Linton 2005). In phase I and II clinical trials in rheumatoid arthritis patients, VX-740 and VX-765 showed anti-inflammatory effects and excellent pharmacokinetic profiles (Strand and Sokolove 2009). However, no additional development was pursued because of the hepatotoxicity induced after long-term exposure in animals (Fischer and Schulze-Osthoff 2005; Wannamaker et al. 2007). According to a recent study, VX-765 alleviated cognitive impairments and the severity of AD (Flores et al. 2018). Additionally, VX-765 alleviated myocardial infarction by preserving ventricular functions in mice (Audia et al. 2018).

### Targeting IL-1β and IL-18

Anakinra is a recombinant interleukin 1 (IL-1) receptor antagonist. A number of clinical trials on the efficacy and safety of anakinra were conducted in gout and rheumatoid arthritis (So et al. 2007). Anakinra can quickly terminate seizures, prevent recurrence, and resolve seizure-related effects on the blood–brain barrier. Clinical trials have identified the effects of interleukin-1 inhibition on vascular and left ventricular function in rheumatoid arthritis patients with coronary artery disease. A phase 2 clinical trial was performed to assess the efficacy and safety of anakinra in patients with Kawasaki disease who failed to respond to a standard treatment. The safety and tolerability of anakinra-mediated blockade of both IL-1α and IL-1β will be evaluated as a strategy to prevent or attenuate coronary artery damage in infants and children with acute Kawasaki disease (Dinarello and van der Meer 2013).

Canakinumab is a human monoclonal antibody that targets IL-1β approved for the treatment of CAPS and is undergoing clinical trials for chronic obstructive pulmonary disease (COPD) and gout. In 2016, a clinical study was performed to investigate the use of canakinumab for the treatment of adult-onset Still's disease (AOSD) (Church and McDermott 2010; Kone-Paut et al. 2011).

Gevokizumab binds with high affinity to IL-1β and has unique allosteric modulating properties. Gevokizumab has a potential to treat patients with a wide variety of inflammatory and other diseases (Cavelti-Weder et al. 2012). Clinical trials of the efficacy and safety of gevokizumab against acne vulgaris were conducted to determine whether gevokizumab was effective for the treatment of moderate to severe acne vulgaris (Fenini et al. 2017).

Rilonacept is an IL-1 inhibitor and is also known as IL-1 Trap (brand name: Arcalyst). Rilonacept is the first FDA-approved drug for the treatment of CAPS, including FCAS and Muckle–Wells syndrome in adults and children (Kone-Paut and Galeotti 2015). In 2012, an FDA Advisory Panel approved rilonacept for the treatment of gout (Schumacher et al. 2012).

GSK1070806 is a humanized monoclonal antibody that blocks the IL-18 protein. From 2009 to 2017, a phase 1 clinical study of GSK1070806 was conducted to test the effect of GSK1070806 in healthy and obese male subjects with normal immune systems to determine the safety of this drug and the rate of metabolism of the antibody. Numerous clinical trials assessed direct inhibition of IL-18 in B-cell non-Hodgkin’s lymphoma and inflammatory bowel disease (IBD) (Mistry et al. 2014).

### Indirect inhibition

#### Auranofin

Auranofin is an Au-containing acetylated carbohydrate complex used for the treatment of rheumatoid arthritis. Monocytes and macrophages in the synovial fluid of RA patients secrete large quantities of IL-1β that lead to the pathophysiological changes associated with RA (Yamada et al. 1999; Han et al. 2008).

Auranofin suppressed LPS-induced NLRP3 and IL-1-related gene expression in J774 cells and primary mouse peritoneal macrophages (Isakov et al. 2014). These results are in line with the known mechanism of action of auranofin by targeting IKK, which is critical for NF-κB activation. In addition, thioredoxin reductase, a redox enzyme responsible for controlling macrophage activation, was another functionally relevant target of auranofin in the blockade of LPS-induced expression of the pro-IL-1β and NLRP3 genes (Isakov et al. 2014). Auranofin inhibited *Propionibacterium acnes*-induced activation of the NLRP3 inflammasome in primary mouse macrophages and human sebocytes resulting in a reduction in inflammatory symptoms in a *P. acnes*-induced mouse acne model (Yang et al. 2020b). Overall, these studies suggest that auranofin inhibits IL-1β secretion by blocking NF-κB activation and subsequent NLRP3/pro-IL-1β transcription.

#### β-Hydroxybutyrate (BHB)

Various stimuli that activate NLRP3-mediated inflammation are specifically inhibited by a ketone body, BHB, which acts as an alternative to glucose under hypoglycemic conditions. In animal models of NLRP3-mediated diseases, such as Muckle–Wells syndrome, familial cold autoinflammatory syndrome, and urate crystal-induced peritonitis, caspase-1 activation and IL-1β secretion were significantly weakened by BHB-complexed nanolipogels and a ketogenic diet, and BHB inhibited K^+^ efflux and ASC aggregation (Youm et al. 2015).

#### Celastrol

Celastrol inhibits NLRP3 inflammasome activation induced by both ATP and nigericin (Lee et al. 2019a). Celastrol blocks the ATP-induced cleavage of procaspase-1 to caspase-1 and pro-IL-1β to mature IL-1β in macrophages. Inhibition of NLRP3 inflammation in macrophages by celastrol is associated with inhibition of tumor cell metastasis (Lee et al. 2019a). Celastrol inhibits ox-LDL-induced NLRP3 inflammasome activation in mesangial cells suggesting a possible contribution of celastrol-mediated NLRP3 inhibition to proliferative glomerular diseases (Sun et al. 2019).

#### Epigallocatechin-3-gallate (EGCG)

EGCG inhibits NLRP3 inflammation by blocking MSU crystal-induced production of caspase-1 (p10) and interleukin-1β in primary mouse macrophages. In an acute gout mouse model, oral administration of EGCG effectively alleviated the symptoms of gout inflammation in mouse foot tissue injected with MSU crystals. EGCG inhibited NLRP3 inflammation via de novo synthesis of mitochondrial DNA and generation of reactive oxygen species in primary mouse macrophages (Lee et al. 2019b).

#### FC11A-2

In THP-1 cells and a mouse model of dextran sulfate sodium (DSS)-induced experimental colitis, 1-ethyl-5-methyl-2-phenyl-1H-benzo[d]imidazole (also known as FC11A-2) inhibited IL-1β/18 release and thus inhibited the NLRP3 inflammasome. FC11A-2 hindered the proximity-induced autocleavage of procaspase-1 to eventually decrease caspase-1 activation via a pathway independent of NF-κB activation (Liu et al. 2013b).

#### Glyburide, 16673–34-0 (JC21), and JC171

Glyburide is a small-molecule inhibitor commonly used to treat T2D. Glyburide was the first compound confirmed to inhibit NLRP3-dependent IL-1β production (Lamkanfi et al. 2009b; Masters et al. 2010). Glyburide inhibits NLRP3 inflammasome activity caused by islet amyloid polypeptide (IAPP), ATP, and nigericin (Masters et al. 2010). Glyburide suppresses ATP-sensitive K + channels downstream of P2X7 receptors and ASC aggregation. Glyburide was effective in preventing endotoxic shock-induced lethality in an animal model of T2D (Lamkanfi et al. 2009a).

16673-34-0 (referred to as JC21) is an intermediate substrate in glyburide synthesis. 16673-34-0 does not affect glucose metabolism due to absence of the cyclohexylurea moiety, which participates in insulin release, present in glyburide. 16673-34-0 inhibits the formation of the NLRP3 inflammasome in mouse macrophages and primary adult rat cardiomyocytes; however, the compound does not affect the AIM2 or NLRC4 inflammasome (Marchetti et al. 2014). Although 16673-34-0 has shown promising activity as an NLRP3 inflammasome inhibitor, its low solubility has been recognized as a problem for further development (Marchetti et al. 2014). Therefore, to increase the polarity of this compound, a new analog (JC171) containing hydroxamic acid on the sulfonamide moiety was developed (Guo et al. 2017). JC171 disrupted the NLRP3/ASC interaction induced by LPS/ATP stimulation in macrophages. JC171 treatment reduced the severity of experimental autoimmune encephalomyelitis (EAE) in a mouse model of multiple sclerosis.

#### JC124

Kuwar et al*.* developed a novel small molecule, JC124, through structural optimization of glyburide (Kuwar et al. 2019). JC124 was designed to prevent the potential hypoglycemic effects of glyburide. Exploration of the potential of JC124 for the treatment of traumatic brain injury (TBI) demonstrated that the compound has significant anti-inflammatory effects on the TBI-affected brain. Treatment with JC124 significantly decreased the expression of NLRP3, ASC, caspase-1, pro-IL-1β, TNFα, and inducible nitric oxide synthase (iNOS). Protective effects of JC124 on TBI were suggested to be due to the activation of the NLRP3 inflammasome and targeting of its downstream neuroinflammatory cascade (Kuwar et al. 2019). JC124 was shown to block ASC aggregation, caspase-1 activation, and IL-1β secretion and thus exert protective effects in the mouse models of acute myocardial infarction (Fulp et al. 2018) and in transgenic AD models (Yin et al. 2018).

#### Licochalcone A

Licochalcone A, a chalconoid isolated from the root of *Glycyrrhiza inflatae*, inhibits *P. acnes*-induced NLRP3 inflammasome activation. Licochalcone A blocks caspase-1 (p10) and IL-1β production in primary mouse macrophages and human SZ95 sebocytes. In addition, Licochalcone A inhibits ASC speck formation and mitochondrial reactive oxygen species. The topical application of licochalcone A to mouse ear skin attenuated *P. acnes*-induced skin inflammation according to the data of histological evaluation, ear thickness measurement, and inflammatory gene expression. Licochalcone A reduced caspase-1 activity and IL-1β production in mouse ears injected with *P. acne* (Yang et al. 2018a). Licochalcone A induced UCP1 expression in adipocytes and inguinal white adipose tissue of high-fat diet-fed mice (Lee et al. 2018). Licochalcone A treatment improved metabolic homeostasis compromised by a high-fat diet and blocked obesity (Lee et al. 2018). Considering the role of the NLRP3 inflammasome in inducing metabolic disorders, the inhibitory effect of licochalcone A on the NLRP3 inflammasome may mediate the blockade of high-fat diet-induced obesity and metabolic disorders by the compound.

#### Sulforaphane

In an acute gout mouse model, oral administration of sulforaphane (SFN) attenuated MSU crystal-induced swelling and neutrophil recruitment thus indicating inhibition of NLRP3 inflammatory activation in the foot tissue. In primary mouse macrophages, SFN inhibited NLRP3 inflammasome activation induced by MSU crystals, adenosine triphosphate, and nigericin independent of the reactive oxygen species pathway. SFN inhibited ligand-independent activation of the NLRP3 inflammasome suggesting that SFN acts directly on the NLRP3 inflammasome complex (Yang et al. 2018b). Oral administration of SFN prevented hepatic steatosis in a high-fat diet-induced mouse NAFLD model, and the effect was mediated by inhibition of the lipid-induced NLRP3 inflammasome in hepatocytes by SFN (Yang et al. 2016). SFN regulates the AMP-activated protein kinase-autophagy axis resulting in the suppression of the NLRP3 inflammasome in hepatocytes (Yang et al. 2016).

In summary, intensive efforts evaluated small molecules and phytochemicals as pharmacological inhibitors of the NLRP3 inflammasome. To date, certain small molecule inhibitors have specific targets, especially those inhibitors that directly bind to the NLRP3 inflammasome components, and some of the inhibitors nonspecifically regulate cellular signaling pathways. It is important to identify the exact mechanism of inhibition of the NLRP3 inflammasome by these small molecules to finally develop high potency pharmacological inhibitors specific to the NLRP3 inflammasome.

## Perspectives

The need for NLRP3-specific treatments will increase because of an increase in the number of individuals affected by inflammatory diseases associated with Western lifestyle and aging population. Many NLRP3 inhibitors that indirectly or directly inhibit the activation of the NLRP3 inflammasome have been developed and reached clinical trials. Nevertheless, no treatment is currently approved by the Food and Drug Administration (FDA) or other agencies. It is critical to evaluate the safety, tolerance, and dose-dependent toxicity of NLRP3 inflammasome modulators for effective treatment. Future investigations will require chemical studies of the structure and phase separation to uncover the basic principles of NLRP3 inflammasome assembly. Studies should also use the advantage of the currently available NLRP3 structure and focus on the development of direct structure-induced inhibitors with improved specificity and efficacy. Continued profiling, restructuring, and improvements in the pharmacokinetic properties of specific NLRP3 inhibitors will facilitate clinical translation in the future to demonstrate the use of precision medicine in inflammasome-related diseases.

## References

[CR1] Abderrazak A, Syrovets T, Couchie D, El Hadri K, Friguet B, Simmet T, Rouis M (2015). NLRP3 inflammasome: from a danger signal sensor to a regulatory node of oxidative stress and inflammatory diseases. Redox Biol.

[CR2] Altaf A, Qu P, Zhao Y, Wang H, Lou D, Niu N (2015). NLRP3 inflammasome in peripheral blood monocytes of acute coronary syndrome patients and its relationship with statins. Coron Artery Dis.

[CR3] Audia JP, Yang XM, Crockett ES, Housley N, Haq EU, O'donnell K, Cohen MV, Downey JM, Alvarez DF,  (2018). Caspase-1 inhibition by VX-765 administered at reperfusion in P2Y12 receptor antagonist-treated rats provides long-term reduction in myocardial infarct size and preservation of ventricular function. Basic Res Cardiol.

[CR4] Bae JY, Lee SW, Shin YH, Lee JH, Jahng JW, Park K (2017). P2X7 receptor and NLRP3 inflammasome activation in head and neck cancer. Oncotarget.

[CR5] Baron L, Gombault A, Fanny M, Villeret B, Savigny F, Guillou N, Panek C, Le Bert M, Lagente V, Rassendren F, Riteau N, Couillin I (2015). The NLRP3 inflammasome is activated by nanoparticles through ATP, ADP and adenosine. Cell Death Dis.

[CR6] Bauernfeind FG, Horvath G, Stutz A, Alnemri ES, Macdonald K, Speert D, Fernandes-Alnemri T, Wu J, Monks BG, Fitzgerald KA, Hornung V, Latz E (2009). Cutting edge: NF-kappaB activating pattern recognition and cytokine receptors license NLRP3 inflammasome activation by regulating NLRP3 expression. J Immunol.

[CR7] Bivik C, Verma D, Winge MC, Lieden A, Bradley M, Rosdahl I, Soderkvist P (2013). Genetic variation in the inflammasome and atopic dermatitis susceptibility. J Invest Dermatol.

[CR8] Booshehri LM, Hoffman HM (2019). CAPS and NLRP3. J Clin Immunol.

[CR10] Brill JM, Mccarty DJ (1964). "Studies on the Nature of Gouty Tophi" by Max Freudweiler, 1899. (an Inflammatory Response to Injected Sodium Urate, 1899). An Abridged Translation, with Comments. Ann Intern Med.

[CR11] Carlstrom M, Ekman AK, Petersson S, Soderkvist P, Enerback C (2012). Genetic support for the role of the NLRP3 inflammasome in psoriasis susceptibility. Exp Dermatol.

[CR204] Cavelti-Weder C, Babians-Brunner A, Keller C, Stahel MA, Kurz-Levin M, Zayed H, Solinger AM, Mandrup-Poulsen T, Dinarello CA, Donath MY (2012) Effects of gevokizumab on glycemia and inflammatory markers in type 2 diabetes. Diabetes Care 35:1654–1662. 10.2337/dc11-2219 10.2337/dc11-2219 PMC3402269 22699287

[CR12] Chen CS, Chang PJ, Lin WY, Huang YC, Ho DR (2013). Evidences of the inflammasome pathway in chronic prostatitis and chronic pelvic pain syndrome in an animal model. Prostate.

[CR13] Chen FF, Tang HY, Yu F, Que CL, Zhou FD, Wang SX, Wang GF, Zhao MH (2019). Renal involvement in a silicosis patient - case report and literature review. Ren Fail.

[CR14] Chen GF, Xu TH, Yan Y, Zhou YR, Jiang Y, Melcher K, Xu HE (2017). Amyloid beta: structure, biology and structure-based therapeutic development. Acta Pharmacol Sin.

[CR15] Church LD, Mcdermott MF (2010). Canakinumab: a human anti-IL-1beta monoclonal antibody for the treatment of cryopyrin-associated periodic syndromes. Expert Rev Clin Immunol.

[CR16] Coll RC, Hill JR, Day CJ, Zamoshnikova A, Boucher D, Massey NL, Chitty JL, Fraser JA, Jennings MP, AaB R, Schroder K (2019). MCC950 directly targets the NLRP3 ATP-hydrolysis motif for inflammasome inhibition. Nat Chem Biol.

[CR17] Coll RC, Robertson AA, Chae JJ, Higgins SC, Munoz-Planillo R, Inserra MC, Vetter I, Dungan LS, Monks BG, Stutz A, Croker DE, Butler MS, Haneklaus M, Sutton CE, Nunez G, Latz E, Kastner DL, Mills KH, Masters SL, Schroder K, Cooper MA, O’neill LA (2015). A small-molecule inhibitor of the NLRP3 inflammasome for the treatment of inflammatory diseases. Nat Med.

[CR18] Cruz CM, Rinna A, Forman HJ, Ventura AL, Persechini PM, Ojcius DM (2007). ATP activates a reactive oxygen species-dependent oxidative stress response and secretion of proinflammatory cytokines in macrophages. J Biol Chem.

[CR19] D'anneo A, Carlisi D, Lauricella M, Puleio R, Martinez R, Di Bella S, Di Marco P, Emanuele S, Di Fiore R, Guercio A, Vento R, Tesoriere G (2013) Parthenolide generates reactive oxygen species and autophagy in MDA-MB231 cells. A soluble parthenolide analogue inhibits tumour growth and metastasis in a xenograft model of breast cancer. Cell Death Dis 4:e891. 10.1038/cddis.2013.415 10.1038/cddis.2013.415 PMC3920954 24176849

[CR20] Dai X, Sayama K, Tohyama M, Shirakata Y, Hanakawa Y, Tokumaru S, Yang L, Hirakawa S, Hashimoto K (2011). Mite allergen is a danger signal for the skin via activation of inflammasome in keratinocytes. J Allergy Clin Immunol.

[CR21] Darakhshan S, Pour AB (2015). Tranilast: a review of its therapeutic applications. Pharmacol Res.

[CR22] Dinarello CA, Van Der Meer JW (2013). Treating inflammation by blocking interleukin-1 in humans. Semin Immunol.

[CR23] Dixon LJ, Berk M, Thapaliya S, Papouchado BG, Feldstein AE (2012). Caspase-1-mediated regulation of fibrogenesis in diet-induced steatohepatitis. Lab Invest.

[CR24] Donath MY, Shoelson SE (2011). Type 2 diabetes as an inflammatory disease. Nat Rev Immunol.

[CR25] Duewell P, Kono H, Rayner KJ, Sirois CM, Vladimer G, Bauernfeind FG, Abela GS, Franchi L, Nunez G, Schnurr M, Espevik T, Lien E, Fitzgerald KA, Rock KL, Moore KJ, Wright SD, Hornung V, Latz E (2010). NLRP3 inflammasomes are required for atherogenesis and activated by cholesterol crystals. Nature.

[CR26] Ekman AK, Verma D, Fredrikson M, Bivik C, Enerback C (2014). Genetic variations of NLRP1: susceptibility in psoriasis. Br J Dermatol.

[CR27] El-Omar EM, Carrington M, Chow WH, Mccoll KE, Bream JH, Young HA, Herrera J, Lissowska J, Yuan CC, Rothman N, Lanyon G, Martin M, Fraumeni JF, Rabkin CS (2000). Interleukin-1 polymorphisms associated with increased risk of gastric cancer. Nature.

[CR28] Ezzedine K, Eleftheriadou V, Whitton M, Van Geel N (2015). Vitiligo. Lancet.

[CR29] Fenini G, Contassot E, French LE (2017). Potential of IL-1, IL-18 and inflammasome inhibition for the treatment of inflammatory skin diseases. Front Pharmacol.

[CR30] Ferrari D, Pizzirani C, Adinolfi E, Lemoli RM, Curti A, Idzko M, Panther E, Di Virgilio F (2006). The P2X7 receptor: a key player in IL-1 processing and release. J Immunol.

[CR31] Fink SL, Cookson BT (2006). Caspase-1-dependent pore formation during pyroptosis leads to osmotic lysis of infected host macrophages. Cell Microbiol.

[CR32] Fischer U, Schulze-Osthoff K (2005). Apoptosis-based therapies and drug targets. Cell Death Differ.

[CR33] Flores J, Noel A, Foveau B, Lynham J, Lecrux C, Leblanc AC (2018). Caspase-1 inhibition alleviates cognitive impairment and neuropathology in an Alzheimer's disease mouse model. Nat Commun.

[CR34] Franchi L, Eigenbrod T, Munoz-Planillo R, Ozkurede U, Kim YG, Arindam C, Gale M, Silverman RH, Colonna M, Akira S, Nunez G (2014). Cytosolic double-stranded RNA activates the NLRP3 inflammasome via MAVS-induced membrane permeabilization and K+ efflux. J Immunol.

[CR35] Franchi L, Warner N, Viani K, Nunez G (2009). Function of Nod-like receptors in microbial recognition and host defense. Immunol Rev.

[CR36] Franklin BS, Bossaller L, De Nardo D, Ratter JM, Stutz A, Engels G, Brenker C, Nordhoff M, Mirandola SR, Al-Amoudi A, Mangan MS, Zimmer S, Monks BG, Fricke M, Schmidt RE, Espevik T, Jones B, Jarnicki AG, Hansbro PM, Busto P, Marshak-Rothstein A, Hornemann S, Aguzzi A, Kastenmuller W, Latz E (2014). The adaptor ASC has extracellular and 'prionoid' activities that propagate inflammation. Nat Immunol.

[CR37] Fulp J, He L, Toldo S, Jiang Y, Boice A, Guo C, Li X, Rolfe A, Sun D, Abbate A, Wang XY, Zhang S (2018). Structural insights of benzenesulfonamide analogues as NLRP3 inflammasome inhibitors: design, synthesis, and biological characterization. J Med Chem.

[CR39] Graham GM, Farrar MD, Cruse-Sawyer JE, Holland KT, Ingham E (2004). Proinflammatory cytokine production by human keratinocytes stimulated with *Propionibacterium acnes* and *P. acnes* GroEL. Br J Dermatol.

[CR40] Green JP, Yu S, Martin-Sanchez F, Pelegrin P, Lopez-Castejon G, Lawrence CB, Brough D (2018). Chloride regulates dynamic NLRP3-dependent ASC oligomerization and inflammasome priming. Proc Natl Acad Sci USA.

[CR41] Grewe M, Walther S, Gyufko K, Czech W, Schopf E, Krutmann J (1995). Analysis of the cytokine pattern expressed in situ in inhalant allergen patch test reactions of atopic dermatitis patients. J Invest Dermatol.

[CR42] Guo C, Fulp JW, Jiang Y, Li X, Chojnacki JE, Wu J, Wang XY, Zhang S (2017). Development and characterization of a hydroxyl-sulfonamide analogue, 5-chloro-N-[2-(4-hydroxysulfamoyl-phenyl)-ethyl]-2-methoxy-benzamide, as a novel NLRP3 inflammasome inhibitor for potential treatment of multiple sclerosis. ACS Chem Neurosci.

[CR43] Gurcel L, Abrami L, Girardin S, Tschopp J, Van Der Goot FG (2006). Caspase-1 activation of lipid metabolic pathways in response to bacterial pore-forming toxins promotes cell survival. Cell.

[CR44] Hafner-Bratkovic I, Susjan P, Lainscek D, Tapia-Abellan A, Cerovic K, Kadunc L, Angosto-Bazarra D, Pelegrin P, Jerala R (2018). NLRP3 lacking the leucine-rich repeat domain can be fully activated via the canonical inflammasome pathway. Nat Commun.

[CR45] Halle A, Hornung V, Petzold GC, Stewart CR, Monks BG, Reinheckel T, Fitzgerald KA, Latz E, Moore KJ, Golenbock DT (2008). The NALP3 inflammasome is involved in the innate immune response to amyloid-beta. Nat Immunol.

[CR46] Han S, Kim K, Kim H, Kwon J, Lee YH, Lee CK, Song Y, Lee SJ, Ha N, Kim K (2008). Auranofin inhibits overproduction of pro-inflammatory cytokines, cyclooxygenase expression and PGE2 production in macrophages. Arch Pharm Res.

[CR47] He H, Jiang H, Chen Y, Ye J, Wang A, Wang C, Liu Q, Liang G, Deng X, Jiang W, Zhou R (2018). Oridonin is a covalent NLRP3 inhibitor with strong anti-inflammasome activity. Nat Commun.

[CR48] He XF, Xu JH, Li G, Li MY, Li LL, Pei Z, Zhang LY, Hu XQ (2020). NLRP3-dependent microglial training impaired the clearance of amyloid-beta and aggravated the cognitive decline in Alzheimer's disease. Cell Death Dis.

[CR49] He Y, Varadarajan S, Munoz-Planillo R, Burberry A, Nakamura Y, Nunez G (2014). 3,4-methylenedioxy-beta-nitrostyrene inhibits NLRP3 inflammasome activation by blocking assembly of the inflammasome. J Biol Chem.

[CR50] Heinrich M, Robles M, West JE, Ortiz De Montellano BR, Rodriguez E (1998). Ethnopharmacology of Mexican asteraceae (Compositae). Annu Rev Pharmacol Toxicol.

[CR51] Heneka MT, Kummer MP, Stutz A, Delekate A, Schwartz S, Vieira-Saecker A, Griep A, Axt D, Remus A, Tzeng TC, Gelpi E, Halle A, Korte M, Latz E, Golenbock DT (2013). NLRP3 is activated in Alzheimer's disease and contributes to pathology in APP/PS1 mice. Nature.

[CR52] Hornung V, Bauernfeind F, Halle A, Samstad EO, Kono H, Rock KL, Fitzgerald KA, Latz E (2008). Silica crystals and aluminum salts activate the NALP3 inflammasome through phagosomal destabilization. Nat Immunol.

[CR53] Huang Y, Jiang H, Chen Y, Wang X, Yang Y, Tao J, Deng X, Liang G, Zhang H, Jiang W, Zhou R (2018). Tranilast directly targets NLRP3 to treat inflammasome-driven diseases. EMBO Mol Med.

[CR54] Isakov E, Weisman-Shomer P, Benhar M (2014). Suppression of the pro-inflammatory NLRP3/interleukin-1beta pathway in macrophages by the thioredoxin reductase inhibitor auranofin. Biochim Biophys Acta.

[CR55] Jiang H, He H, Chen Y, Huang W, Cheng J, Ye J, Wang A, Tao J, Wang C, Liu Q, Jin T, Jiang W, Deng X, Zhou R (2017). Identification of a selective and direct NLRP3 inhibitor to treat inflammatory disorders. J Exp Med.

[CR56] Johansen C, Moeller K, Kragballe K, Iversen L (2007). The activity of caspase-1 is increased in lesional psoriatic epidermis. J Invest Dermatol.

[CR57] Joosten LA, Netea MG, Fantuzzi G, Koenders MI, Helsen MM, Sparrer H, Pham CT, Van Der Meer JW, Dinarello CA, Van Den Berg WB (2009). Inflammatory arthritis in caspase 1 gene-deficient mice: contribution of proteinase 3 to caspase 1-independent production of bioactive interleukin-1beta. Arthritis Rheum.

[CR58] Juliana C, Fernandes-Alnemri T, Wu J, Datta P, Solorzano L, Yu JW, Meng R, Quong AA, Latz E, Scott CP, Alnemri ES (2010). Anti-inflammatory compounds parthenolide and Bay 11–7082 are direct inhibitors of the inflammasome. J Biol Chem.

[CR59] Kadota S, Basnet P, Ishii E, Tamura T, Namba T (1997). Antibacterial activity of trichorabdal A from Rabdosia trichocarpa against Helicobacter pylori. Zentralbl Bakteriol.

[CR60] Kahlenberg JM, Kaplan MJ (2014). The inflammasome and lupus: another innate immune mechanism contributing to disease pathogenesis?. Curr Opin Rheumatol.

[CR62] Kastbom A, Verma D, Eriksson P, Skogh T, Wingren G, Soderkvist P (2008). Genetic variation in proteins of the cryopyrin inflammasome influences susceptibility and severity of rheumatoid arthritis (the Swedish TIRA project). Rheumatology (Oxford).

[CR63] Katsnelson MA, Lozada-Soto KM, Russo HM, Miller BA, Dubyak GR (2016). NLRP3 inflammasome signaling is activated by low-level lysosome disruption but inhibited by extensive lysosome disruption: roles for K+ efflux and Ca2+ influx. Am J Physiol Cell Physiol.

[CR64] Katsnelson MA, Rucker LG, Russo HM, Dubyak GR (2015). K+ efflux agonists induce NLRP3 inflammasome activation independently of Ca2+ signaling. J Immunol.

[CR65] Keane RW, Dietrich WD, De Rivero Vaccari JP (2018). Inflammasome Proteins As Biomarkers of Multiple Sclerosis. Front Neurol.

[CR66] Kelley N, Jeltema D, Duan Y, He Y (2019). The NLRP3 Inflammasome: An Overview of Mechanisms of Activation and Regulation. Int J Mol Sci.

[CR67] Kerr ID, Lee JH, Farady CJ, Marion R, Rickert M, Sajid M, Pandey KC, Caffrey CR, Legac J, Hansell E, Mckerrow JH, Craik CS, Rosenthal PJ, Brinen LS (2009). Vinyl sulfones as antiparasitic agents and a structural basis for drug design. J Biol Chem.

[CR68] Kistowska M, Gehrke S, Jankovic D, Kerl K, Fettelschoss A, Feldmeyer L, Fenini G, Kolios A, Navarini A, Ganceviciene R, Schauber J, Contassot E, French LE (2014). IL-1beta drives inflammatory responses to propionibacterium acnes in vitro and in vivo. J Invest Dermatol.

[CR203] Kone-Paut I, Galeotti C (2015) Current treatment recommendations and considerations for cryopyrin-associated periodic syndrome. Expert Rev Clin Immunol 11:1083–1092. 10.1586/1744666X.2015.1077702 10.1586/1744666X.2015.1077702 26312542

[CR69] Kone-Paut I, Lachmann HJ, Kuemmerle-Deschner JB, Hachulla E, Leslie KS, Mouy R, Ferreira A, Lheritier K, Patel N, Preiss R, Hawkins PN, Canakinumab In CSG (2011). Sustained remission of symptoms and improved health-related quality of life in patients with cryopyrin-associated periodic syndrome treated with canakinumab: results of a double-blind placebo-controlled randomized withdrawal study. Arthritis Res Ther.

[CR71] Kumar H, Kumagai Y, Tsuchida T, Koenig PA, Satoh T, Guo Z, Jang MH, Saitoh T, Akira S, Kawai T (2009). Involvement of the NLRP3 inflammasome in innate and humoral adaptive immune responses to fungal beta-glucan. J Immunol.

[CR72] Kuwar R, Rolfe A, Di L, Xu H, He L, Jiang Y, Zhang S, Sun D (2019). A novel small molecular NLRP3 inflammasome inhibitor alleviates neuroinflammatory response following traumatic brain injury. J Neuroinflammation.

[CR73] Lamkanfi M, Malireddi RK, Kanneganti TD (2009). Fungal zymosan and mannan activate the cryopyrin inflammasome. J Biol Chem.

[CR74] Lamkanfi M, Mueller JL, Vitari AC, Misaghi S, Fedorova A, Deshayes K, Lee WP, Hoffman HM, Dixit VM (2009). Glyburide inhibits the Cryopyrin/Nalp3 inflammasome. J Cell Biol.

[CR75] Landis RC, Haskard DO (2001). Pathogenesis of crystal-induced inflammation. Curr Rheumatol Rep.

[CR76] Larsen CM, Faulenbach M, Vaag A, Volund A, Ehses JA, Seifert B, Mandrup-Poulsen T, Donath MY (2007). Interleukin-1-receptor antagonist in type 2 diabetes mellitus. N Engl J Med.

[CR77] Lee HE, Lee JY, Yang G, Kang HC, Cho YY, Lee HS, Lee JY (2019). Inhibition of NLRP3 inflammasome in tumor microenvironment leads to suppression of metastatic potential of cancer cells. Sci Rep.

[CR78] Lee HE, Yang G, Han SH, Lee JH, An TJ, Jang JK, Lee JY (2018). Anti-obesity potential of Glycyrrhiza uralensis and licochalcone A through induction of adipocyte browning. Biochem Biophys Res Commun.

[CR79] Lee HE, Yang G, Kim ND, Jeong S, Jung Y, Choi JY, Park HH, Lee JY (2016). Targeting ASC in NLRP3 inflammasome by caffeic acid phenethyl ester: a novel strategy to treat acute gout. Sci Rep.

[CR80] Lee HE, Yang G, Park YB, Kang HC, Cho YY, Lee HS, Lee JY (2019). Epigallocatechin-3-gallate prevents acute gout by suppressing NLRP3 inflammasome activation and mitochondrial DNA synthesis. Molecules.

[CR81] Lee HM, Kim JJ, Kim HJ, Shong M, Ku BJ, Jo EK (2013). Upregulated NLRP3 inflammasome activation in patients with type 2 diabetes. Diabetes.

[CR82] Lewis J, Dickson DW (2016). Propagation of tau pathology: hypotheses, discoveries, and yet unresolved questions from experimental and human brain studies. Acta Neuropathol.

[CR83] Li S, Kang P, Zhang W, Jian Z, Zhang Q, Yi X, Guo S, Guo W, Shi Q, Li B, He Y, Song P, Liu L, Li K, Wang G, Gao T, Li C (2020). Activated NLR family pyrin domain containing 3 (NLRP3) inflammasome in keratinocytes promotes cutaneous T-cell response in patients with vitiligo. J Allergy Clin Immunol.

[CR84] Li ZJ, Choi DK, Sohn KC, Seo MS, Lee HE, Lee Y, Seo YJ, Lee YH, Shi G, Zouboulis CC, Kim CD, Lee JH, Im M (2014). Propionibacterium acnes activates the NLRP3 inflammasome in human sebocytes. J Invest Dermatol.

[CR201] Linton SD (2005) Caspase inhibitors: a pharmaceutical industry perspective. Curr Top Med Chem 5:1697–1717. 10.2174/156802605775009720 10.2174/156802605775009720 16375749

[CR85] Liu A, Gao X, Zhang Q, Cui L (2013). Cathepsin B inhibition attenuates cardiac dysfunction and remodeling following myocardial infarction by inhibiting the NLRP3 pathway. Mol Med Rep.

[CR86] Liu W, Guo W, Wu J, Luo Q, Tao F, Gu Y, Shen Y, Li J, Tan R, Xu Q, Sun Y (2013). A novel benzo[d]imidazole derivate prevents the development of dextran sulfate sodium-induced murine experimental colitis via inhibition of NLRP3 inflammasome. Biochem Pharmacol.

[CR87] Lonnemann N, Hosseini S, Marchetti C, Skouras DB, Stefanoni D, D’alessandro A, Dinarello CA, Korte M (2020). The NLRP3 inflammasome inhibitor OLT1177 rescues cognitive impairment in a mouse model of Alzheimer's disease. Proc Natl Acad Sci USA.

[CR89] Losy J, Niezgoda A (2001). IL-18 in patients with multiple sclerosis. Acta Neurol Scand.

[CR90] Lu A, Li H, Niu J, Wu S, Xue G, Yao X, Guo Q, Wan N, Abliz P, Yang G, An L, Meng G (2017). Hyperactivation of the NLRP3 inflammasome in myeloid cells leads to severe organ damage in experimental lupus. J Immunol.

[CR91] Ma T, Thiagarajah JR, Yang H, Sonawane ND, Folli C, Galietta LJ, Verkman AS (2002). Thiazolidinone CFTR inhibitor identified by high-throughput screening blocks cholera toxin-induced intestinal fluid secretion. J Clin Invest.

[CR92] Malhotra S, Costa C, Eixarch H, Keller CW, Amman L, Martinez-Banaclocha H, Midaglia L, Sarro E, Machin-Diaz I, Villar LM, Trivino JC, Oliver-Martos B, Parlade LN, Calvo-Barreiro L, Matesanz F, Vandenbroeck K, Urcelay E, Martinez-Gines ML, Tejeda-Velarde A, Fissolo N, Castillo J, Sanchez A, AaB R, Clemente D, Prinz M, Pelegrin P, Lunemann JD, Espejo C, Montalban X, Comabella M (2020). NLRP3 inflammasome as prognostic factor and therapeutic target in primary progressive multiple sclerosis patients. Brain.

[CR93] Marchetti C, Chojnacki J, Toldo S, Mezzaroma E, Tranchida N, Rose SW, Federici M, Van Tassell BW, Zhang S, Abbate A (2014). A novel pharmacologic inhibitor of the NLRP3 inflammasome limits myocardial injury after ischemia-reperfusion in the mouse. J Cardiovasc Pharmacol.

[CR94] Marchetti C, Swartzwelter B, Gamboni F, Neff CP, Richter K, Azam T, Carta S, Tengesdal I, Nemkov T, Dalessandro A, Henry C, Jones GS, Goodrich SA, St Laurent JP, Jones TM, Scribner CL, Barrow RB, Altman RD, Skouras DB, Gattorno M, Grau V, Janciauskiene S, Rubartelli A, LaB Joosten, Dinarello CA (2018). OLT1177, a beta-sulfonyl nitrile compound, safe in humans, inhibits the NLRP3 inflammasome and reverses the metabolic cost of inflammation. Proc Natl Acad Sci USA.

[CR95] Marchetti C, Swartzwelter B, Koenders MI, Azam T, Tengesdal IW, Powers N, De Graaf DM, Dinarello CA, LaB J (2018). NLRP3 inflammasome inhibitor OLT1177 suppresses joint inflammation in murine models of acute arthritis. Arthritis Res Ther.

[CR96] Martinon F, Petrilli V, Mayor A, Tardivel A, Tschopp J (2006). Gout-associated uric acid crystals activate the NALP3 inflammasome. Nature.

[CR97] Masters CL, Selkoe DJ (2012). Biochemistry of amyloid beta-protein and amyloid deposits in Alzheimer disease. Cold Spring Harb Perspect Med.

[CR98] Masters SL, Dunne A, Subramanian SL, Hull RL, Tannahill GM, Sharp FA, Becker C, Franchi L, Yoshihara E, Chen Z, Mullooly N, Mielke LA, Harris J, Coll RC, Mills KH, Mok KH, Newsholme P, Nunez G, Yodoi J, Kahn SE, Lavelle EC, O'neill LA (2010) Activation of the NLRP3 inflammasome by islet amyloid polypeptide provides a mechanism for enhanced IL-1beta in type 2 diabetes. Nat Immunol 11**:**897–904. 10.1038/ni.1935 10.1038/ni.1935 PMC3103663 20835230

[CR99] Masters SL, Simon A, Aksentijevich I, Kastner DL (2009). Horror autoinflammaticus: the molecular pathophysiology of autoinflammatory disease (*). Annu Rev Immunol.

[CR100] Mathews RJ, Robinson JI, Battellino M, Wong C, Taylor JC, Biologics in Rheumatoid Arthritis G, Genomics Study S, Eyre S, Churchman SM, Wilson AG, Isaacs JD, Hyrich K, Barton A, Plant D, Savic S, Cook GP, Sarzi-Puttini P, Emery P, Barrett JH, Morgan AW, Mcdermott MF (2014) Evidence of NLRP3-inflammasome activation in rheumatoid arthritis (RA); genetic variants within the NLRP3-inflammasome complex in relation to susceptibility to RA and response to anti-TNF treatment. Ann Rheum Dis 73**:**1202–1210. 10.1136/annrheumdis-2013-203276 10.1136/annrheumdis-2013-203276 23687262

[CR101] Mensa-Vilaro A, Teresa Bosque M, Magri G, Honda Y, Martinez-Banaclocha H, Casorran-Berges M, Sintes J, Gonzalez-Roca E, Ruiz-Ortiz E, Heike T, Martinez-Garcia JJ, Baroja-Mazo A, Cerutti A, Nishikomori R, Yague J, Pelegrin P, Delgado-Beltran C, Arostegui JI (2016). Brief Report: late-onset cryopyrin-associated periodic syndrome due to myeloid-restricted somatic NLRP3 mosaicism. Arthritis Rheumatol.

[CR102] Menu P, Vince JE (2011). The NLRP3 inflammasome in health and disease: the good, the bad and the ugly. Clin Exp Immunol.

[CR103] Mistry P, Reid J, Pouliquen I, Mchugh S, Abberley L, Dewall S, Taylor A, Tong X, Rocha Del Cura M, Mckie E (2014). Safety, tolerability, pharmacokinetics, and pharmacodynamics of single-dose antiinterleukin- 18 mAb GSK1070806 in healthy and obese subjects. Int J Clin Pharmacol Ther.

[CR104] Moossavi M, Parsamanesh N, Bahrami A, Atkin SL, Sahebkar A (2018). Role of the NLRP3 inflammasome in cancer. Mol Cancer.

[CR105] Morandini AC, Savio LE, Coutinho-Silva R (2014). The role of P2X7 receptor in infectious inflammatory diseases and the influence of ectonucleotidases. Biomed J.

[CR106] Muckle TJ, Wellsm (1962) Urticaria, deafness, and amyloidosis: a new heredo-familial syndrome. Q J Med 31**:**235-248. 14476827

[CR107] Munoz-Planillo R, Kuffa P, Martinez-Colon G, Smith BL, Rajendiran TM, Nunez G (2013). K(+) efflux is the common trigger of NLRP3 inflammasome activation by bacterial toxins and particulate matter. Immunity.

[CR108] Murakami T, Ockinger J, Yu J, Byles V, Mccoll A, Hofer AM, Horng T (2012). Critical role for calcium mobilization in activation of the NLRP3 inflammasome. Proc Natl Acad Sci U S A.

[CR109] Niebuhr M, Baumert K, Heratizadeh A, Satzger I, Werfel T (2014). Impaired NLRP3 inflammasome expression and function in atopic dermatitis due to Th2 milieu. Allergy.

[CR110] Park S, Juliana C, Hong S, Datta P, Hwang I, Fernandes-Alnemri T, Yu JW, Alnemri ES (2013). The mitochondrial antiviral protein MAVS associates with NLRP3 and regulates its inflammasome activity. J Immunol.

[CR111] Platten M, Ho PP, Youssef S, Fontoura P, Garren H, Hur EM, Gupta R, Lee LY, Kidd BA, Robinson WH, Sobel RA, Selley ML, Steinman L (2005). Treatment of autoimmune neuroinflammation with a synthetic tryptophan metabolite. Science.

[CR112] Ridker PM, Thuren T, Zalewski A, Libby P (2011). Interleukin-1beta inhibition and the prevention of recurrent cardiovascular events: rationale and design of the Canakinumab Anti-inflammatory Thrombosis Outcomes Study (CANTOS). Am Heart J.

[CR113] Rubartelli A (2012). Redox control of NLRP3 inflammasome activation in health and disease. J Leukoc Biol.

[CR114] Sanchez-Fernandez A, Skouras DB, Dinarello CA, Lopez-Vales R (2019). OLT1177 (Dapansutrile), a selective NLRP3 inflammasome inhibitor, ameliorates experimental autoimmune encephalomyelitis pathogenesis. Front Immunol.

[CR115] Sandanger O, Ranheim T, Vinge LE, Bliksoen M, Alfsnes K, Finsen AV, Dahl CP, Askevold ET, Florholmen G, Christensen G, Fitzgerald KA, Lien E, Valen G, Espevik T, Aukrust P, Yndestad A (2013). The NLRP3 inflammasome is up-regulated in cardiac fibroblasts and mediates myocardial ischaemia-reperfusion injury. Cardiovasc Res.

[CR116] Schumacher HR, Evans RR, Saag KG, Clower J, Jennings W, Weinstein SP, Yancopoulos GD, Wang J, Terkeltaub R (2012). Rilonacept (interleukin-1 trap) for prevention of gout flares during initiation of uric acid-lowering therapy: results from a phase III randomized, double-blind, placebo-controlled, confirmatory efficacy study. Arthritis Care Res (Hoboken).

[CR117] Sebastian-Valverde M, Pasinetti GM (2020). The NLRP3 inflammasome as a critical actor in the inflammaging process. Cells.

[CR118] Sha W, Mitoma H, Hanabuchi S, Bao M, Weng L, Sugimoto N, Liu Y, Zhang Z, Zhong J, Sun B, Liu YJ (2014). Human NLRP3 inflammasome senses multiple types of bacterial RNAs. Proc Natl Acad Sci USA.

[CR119] Shimada K, Crother TR, Karlin J, Dagvadorj J, Chiba N, Chen S, Ramanujan VK, Wolf AJ, Vergnes L, Ojcius DM, Rentsendorj A, Vargas M, Guerrero C, Wang Y, Fitzgerald KA, Underhill DM, Town T, Arditi M (2012). Oxidized mitochondrial DNA activates the NLRP3 inflammasome during apoptosis. Immunity.

[CR121] So A, De Smedt T, Revaz S, Tschopp J (2007). A pilot study of IL-1 inhibition by anakinra in acute gout. Arthritis Res Ther.

[CR122] Soares JL, Oliveira EM, Pontillo A (2019). Variants in NLRP3 and NLRC4 inflammasome associate with susceptibility and severity of multiple sclerosis. Mult Scler Relat Disord.

[CR123] Stienstra R, Joosten LA, Koenen T, Van Tits B, Van Diepen JA, Van Den Berg SA, Rensen PC, Voshol PJ, Fantuzzi G, Hijmans A, Kersten S, Muller M, Van Den Berg WB, Van Rooijen N, Wabitsch M, Kullberg BJ, Van Der Meer JW, Kanneganti T, Tack CJ, Netea MG (2010). The inflammasome-mediated caspase-1 activation controls adipocyte differentiation and insulin sensitivity. Cell Metab.

[CR124] Strand V, Sokolove J (2009). Randomized controlled trial design in rheumatoid arthritis: the past decade. Arthritis Res Ther.

[CR125] Stych B, Dobrovolny D (2008). Familial cold auto-inflammatory syndrome (FCAS): characterization of symptomatology and impact on patients' lives. Curr Med Res Opin.

[CR126] Subramanian N, Natarajan K, Clatworthy MR, Wang Z, Germain RN (2013). The adaptor MAVS promotes NLRP3 mitochondrial localization and inflammasome activation. Cell.

[CR127] Sun Z, Li Y, Qian Y, Wu M, Huang S, Zhang A, Zhang Y, Jia Z (2019). Celastrol attenuates ox-LDL-induced mesangial cell proliferation via suppressing NLRP3 inflammasome activation. Cell Death Discov.

[CR128] Takahashi M (2014). NLRP3 inflammasome as a novel player in myocardial infarction. Int Heart J.

[CR129] Takeuchi O, Akira S (2010). Pattern recognition receptors and inflammation. Cell.

[CR130] Tang T, Lang X, Xu C, Wang X, Gong T, Yang Y, Cui J, Bai L, Wang J, Jiang W, Zhou R (2017). CLICs-dependent chloride efflux is an essential and proximal upstream event for NLRP3 inflammasome activation. Nat Commun.

[CR131] Tavera Busso I, Mateos AC, Gonzalez Peroni A, Graziani NS, Carreras HA (2020). Hepatic alterations associated with fine particulate matter exposure. Toxicol Res.

[CR132] Tilg H, Moschen AR (2010). Evolution of inflammation in nonalcoholic fatty liver disease: the multiple parallel hits hypothesis. Hepatology.

[CR133] Tiniakos DG, Vos MB, Brunt EM (2010). Nonalcoholic fatty liver disease: pathology and pathogenesis. Annu Rev Pathol.

[CR134] Toldo S, Abbate A (2018). The NLRP3 inflammasome in acute myocardial infarction. Nat Rev Cardiol.

[CR135] Triantafilou K, Hughes TR, Triantafilou M, Morgan BP (2013). The complement membrane attack complex triggers intracellular Ca2+ fluxes leading to NLRP3 inflammasome activation. J Cell Sci.

[CR136] Tsai PY, Ka SM, Chang JM, Chen HC, Shui HA, Li CY, Hua KF, Chang WL, Huang JJ, Yang SS, Chen A (2011). Epigallocatechin-3-gallate prevents lupus nephritis development in mice via enhancing the Nrf2 antioxidant pathway and inhibiting NLRP3 inflammasome activation. Free Radic Biol Med.

[CR137] Van Den Boorn JG, Jakobs C, Hagen C, Renn M, Luiten RM, Melief CJ, Tuting T, Garbi N, Hartmann G, Hornung V (2016). Inflammasome-dependent induction of adaptive NK cell memory. Immunity.

[CR138] Van Tassell BW, Canada J, Carbone S, Trankle C, Buckley L, Oddi Erdle C, Abouzaki NA, Dixon D, Kadariya D, Christopher S, Schatz A, Regan J, Viscusi M, Del Buono M, Melchior R, Mankad P, Lu J, Sculthorpe R, Biondi-Zoccai G, Lesnefsky E, Arena R, Abbate A (2017). Interleukin-1 blockade in recently decompensated systolic heart failure: results from REDHART (Recently Decompensated Heart Failure Anakinra Response Trial). Circ Heart Fail.

[CR139] Vandanmagsar B, Youm YH, Ravussin A, Galgani JE, Stadler K, Mynatt RL, Ravussin E, Stephens JM, Dixit VD (2011). The NLRP3 inflammasome instigates obesity-induced inflammation and insulin resistance. Nat Med.

[CR140] Venegas C, Kumar S, Franklin BS, Dierkes T, Brinkschulte R, Tejera D, Vieira-Saecker A, Schwartz S, Santarelli F, Kummer MP, Griep A, Gelpi E, Beilharz M, Riedel D, Golenbock DT, Geyer M, Walter J, Latz E, Heneka MT (2017). Microglia-derived ASC specks cross-seed amyloid-beta in Alzheimer's disease. Nature.

[CR141] Vidmar L, Maver A, Drulovic J, Sepcic J, Novakovic I, Ristic S, Sega S, Peterlin B (2019). Multiple Sclerosis patients carry an increased burden of exceedingly rare genetic variants in the inflammasome regulatory genes. Sci Rep.

[CR142] Voet S, Prinz M, Van Loo G (2019). Microglia in central nervous system inflammation and multiple sclerosis pathology. Trends Mol Med.

[CR143] Wang Y, Kong H, Zeng X, Liu W, Wang Z, Yan X, Wang H, Xie W (2016). Activation of NLRP3 inflammasome enhances the proliferation and migration of A549 lung cancer cells. Oncol Rep.

[CR144] Wannamaker W, Davies R, Namchuk M, Pollard J, Ford P, Ku G, Decker C, Charifson P, Weber P, Germann UA, Kuida K, Randle JC (2007). (S)-1-((S)-2-{[1-(4-amino-3-chloro-phenyl)-methanoyl]-amino}-3,3-dimethyl-butanoy l)-pyrrolidine-2-carboxylic acid ((2R,3S)-2-ethoxy-5-oxo-tetrahydro-furan-3-yl)-amide (VX-765), an orally available selective interleukin (IL)-converting enzyme/caspase-1 inhibitor, exhibits potent anti-inflammatory activities by inhibiting the release of IL-1beta and IL-18. J Pharmacol Exp Ther.

[CR145] Wen H, Gris D, Lei Y, Jha S, Zhang L, Huang MT, Brickey WJ, Ting JP (2011). Fatty acid-induced NLRP3-ASC inflammasome activation interferes with insulin signaling. Nat Immunol.

[CR200] Weichand B, Popp R, Dziumbla S, Mora J, Strack E, Elwakeel E, Frank AC, Scholich K, Pierre S, Syed SN, Olesch C, Ringleb J, Oren B, Doring C, Savai R, Jung M, Von Knethen A, Levkau B, Fleming I, Weigert A, Brune B (2017) S1PR1 on tumor-associated macrophages promotes lymphangiogenesis and metastasis via NLRP3/IL-1beta. J Exp Med 214:2695–2713. 10.1084/jem.20160392 10.1084/jem.20160392 PMC5584110 28739604

[CR146] Westman PC, Lipinski MJ, Luger D, Waksman R, Bonow RO, Wu E, Epstein SE (2016). Inflammation as a driver of adverse left ventricular remodeling after acute myocardial infarction. J Am Coll Cardiol.

[CR147] Wree A, Mcgeough MD, Pena CA, Schlattjan M, Li H, Inzaugarat ME, Messer K, Canbay A, Hoffman HM, Feldstein AE (2014). NLRP3 inflammasome activation is required for fibrosis development in NAFLD. J Mol Med (Berl).

[CR148] Xing Y, Yao X, Li H, Xue G, Guo Q, Yang G, An L, Zhang Y, Meng G (2017). Cutting edge: TRAF6 mediates TLR/IL-1R signaling-induced nontranscriptional priming of the NLRP3 inflammasome. J Immunol.

[CR149] Yamada R, Sano H, Hla T, Hashiramoto A, Fukui W, Miyazaki S, Kohno M, Tsubouchi Y, Kusaka Y, Kondo M (1999). Auranofin inhibits interleukin-1beta-induced transcript of cyclooxygenase-2 on cultured human synoviocytes. Eur J Pharmacol.

[CR150] Yan Y, Jiang W, Spinetti T, Tardivel A, Castillo R, Bourquin C, Guarda G, Tian Z, Tschopp J, Zhou R (2013). Omega-3 fatty acids prevent inflammation and metabolic disorder through inhibition of NLRP3 inflammasome activation. Immunity.

[CR151] Yang G, Lee HE, Lee JY (2016). A pharmacological inhibitor of NLRP3 inflammasome prevents non-alcoholic fatty liver disease in a mouse model induced by high fat diet. Sci Rep.

[CR152] Yang G, Lee HE, Moon SJ, Ko KM, Koh JH, Seok JK, Min JK, Heo TH, Kang HC, Cho YY, Lee HS, Fitzgerald KA, Lee JY (2020). Direct binding to NLRP3 pyrin domain as a novel strategy to prevent NLRP3-driven inflammation and Gouty Arthritis. Arthritis Rheumatol.

[CR153] Yang G, Lee HE, Yeon SH, Kang HC, Cho YY, Lee HS, Zouboulis CC, Han SH, Lee JH, Lee JY (2018). Licochalcone A attenuates acne symptoms mediated by suppression of NLRP3 inflammasome. Phytother Res.

[CR154] Yang G, Lee SJ, Kang HC, Cho YY, Lee HS, Zouboulis CC, Han SH, Ma KH, Jang JK, Lee JY (2020). Repurposing auranofin, an anti-rheumatic gold compound, to treat acne vulgaris by targeting the NLRP3 inflammasome. Biomol Ther (Seoul).

[CR155] Yang G, Yeon SH, Lee HE, Kang HC, Cho YY, Lee HS, Lee JY (2018). Suppression of NLRP3 inflammasome by oral treatment with sulforaphane alleviates acute gouty inflammation. Rheumatology (Oxford).

[CR156] Yang Q, Yu C, Yang Z, Wei Q, Mu K, Zhang Y, Zhao W, Wang X, Huai W, Han L (2014). Deregulated NLRP3 and NLRP1 inflammasomes and their correlations with disease activity in systemic lupus erythematosus. J Rheumatol.

[CR157] Yaron JR, Gangaraju S, Rao MY, Kong X, Zhang L, Su F, Tian Y, Glenn HL, Meldrum DR (2015). K(+) regulates Ca(2+) to drive inflammasome signaling: dynamic visualization of ion flux in live cells. Cell Death Dis.

[CR158] Yeon SH, Yang G, Lee HE, Lee JY (2017). Oxidized phosphatidylcholine induces the activation of NLRP3 inflammasome in macrophages. J Leukoc Biol.

[CR159] Yin J, Zhao F, Chojnacki JE, Fulp J, Klein WL, Zhang S, Zhu X (2018). NLRP3 inflammasome inhibitor ameliorates amyloid pathology in a mouse model of Alzheimer's Disease. Mol Neurobiol.

[CR160] Youm YH, Nguyen KY, Grant RW, Goldberg EL, Bodogai M, Kim D, D’agostino D, Planavsky N, Lupfer C, Kanneganti TD, Kang S, Horvath TL, Fahmy TM, Crawford PA, Biragyn A, Alnemri E, Dixit VD (2015). The ketone metabolite beta-hydroxybutyrate blocks NLRP3 inflammasome-mediated inflammatory disease. Nat Med.

[CR161] Zaki MH, Boyd KL, Vogel P, Kastan MB, Lamkanfi M, Kanneganti TD (2010). The NLRP3 inflammasome protects against loss of epithelial integrity and mortality during experimental colitis. Immunity.

[CR162] Zhang Q, Fan HW, Zhang JZ, Wang YM, Xing HJ (2015). NLRP3 rs35829419 polymorphism is associated with increased susceptibility to multiple diseases in humans. Genet Mol Res.

[CR163] Zhang SH, Reddick RL, Piedrahita JA, Maeda N (1992). Spontaneous hypercholesterolemia and arterial lesions in mice lacking apolipoprotein E. Science.

[CR164] Zhou R, Yazdi AS, Menu P, Tschopp J (2011). A role for mitochondria in NLRP3 inflammasome activation. Nature.

